# Non-destructive spectroscopic evaluation of Al substitution and its role in the pink colouration of sugilite aggregates

**DOI:** 10.1039/d6ra04575h

**Published:** 2026-08-07

**Authors:** Pengyu Li, Minghong Shi, Ye Yuan, Mudan Xu, Jiayang Han, Meilin Zhu, Jie Hu, Ying Guo

**Affiliations:** a School of Gemmology, China University of Geosciences Beijing 100083 China guoying@cugb.edu.cn; b School of Design and Innovation, Shenzhen Technology University Shenzhen 518118 China

## Abstract

Sugilite aggregates display a wide range of purple to pink-purple colours, yet the mechanism responsible for pink colouration remains poorly understood. In this study, 100 sugilite aggregate samples were investigated using colourimetry, FTIR, Raman, UV-vis spectroscopy, XRF, and EPMA to establish a non-destructive spectroscopic approach for evaluating Al substitution and clarifying its influence on colour variation. Systematic blue shifts of characteristic FTIR and Raman peaks were observed with increasing Al content and showed strong correlations with the Al_2_O_3_/(Fe_2_O_3_ + Mn_2_O_3_) ratio, indicating that the observed Al variation is predominantly associated with substitution of Al^3+^ for Fe^3+^ and Mn^3+^ at the octahedral A site, accompanied by local structural contraction. UV-vis spectroscopy confirms that Mn^3+^ is the principal chromophore responsible for the purple colouration of sugilite aggregates, while increasing Al substitution modifies the crystal-field environment of Mn^3+^ and causes a blue shift of the main absorption band, driving the colour transition from purple toward pink. In addition, clustering analysis based on the CIE 1976 L*a*b* colour system established four colour grades for sugilite aggregates. These results provide new insights into the crystal-chemical mechanism of pink colouration and demonstrate the potential of non-destructive spectroscopy for evaluating colour-related compositional variations in sugilite aggregates.

## Abstract 2

Non-destructive spectroscopy combined with colourimetry reveals that Al^3+^ substitution for Fe^3+^/Mn^3+^ at the octahedral A site contracts the local structure, modifies the Mn^3+^ crystal field, blue-shifts the 560 nm band, and drives sugilite from purple to pink.

## Graphical abstract

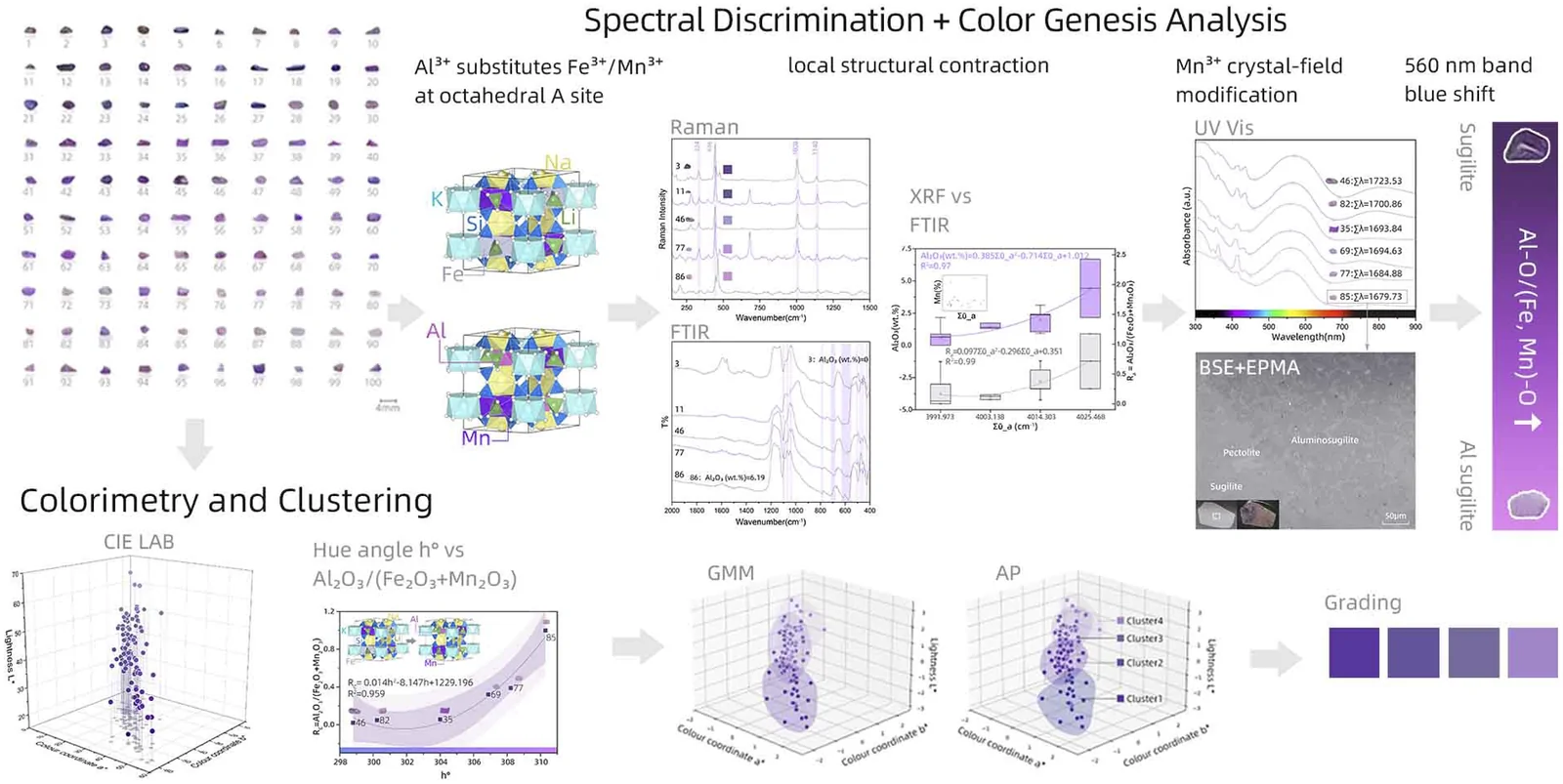

## Introduction

Sugilite is a rare cyclosilicate mineral belonging to the osumilite–milarite group with the general formula (NaK(Fe^3+^, Mn^3+^, Al^3+^)_2_Li_3_Si_12_O_30_).^1–3^ It commonly occurs as polycrystalline aggregates formed through alkali-rich hydrothermal metasomatism in manganese-bearing skarn deposits, particularly in South Africa.^4–6^ Due to their vivid purple to pink-purple appearance, sugilite aggregates have become increasingly important in the gemstone and jade market, especially in East Asia.^7,8^ Among various quality factors, colour is considered the most important determinant of commercial value.

Previous studies generally agree that the characteristic purple colouration of sugilite is mainly caused by Mn^3+^, which produces a broad absorption band in the yellow-green region of the visible spectrum.^5,6,9–11^ However, sugilite aggregates exhibit substantial colour variation, ranging from deep purple to pink-purple and pale pink tones. In particular, high-quality pink sugilite aggregates are highly valued commercially, yet the crystal-chemical mechanism responsible for the pink colouration remains poorly understood.

Several studies have suggested that compositional variations, especially involving Al, Fe, and Mn, may influence the colour of sugilite.^6,12,13^ Nevertheless, these investigations were primarily based on destructive analytical methods such as electron probe microanalysis, which are unsuitable for rapid gemmological testing and commercial applications. More importantly, the relationship between Al substitution, crystal structure modification, and optical absorption behaviour has not yet been systematically clarified. Consequently, a reliable non-destructive method for evaluating colour-related compositional variations in sugilite aggregates is still lacking.

Vibrational spectroscopy provides an effective approach for investigating crystal-chemical variations in silicate minerals. Changes in cation substitution can modify local bonding environments, resulting in systematic shifts in infrared and Raman vibrational frequencies.^14–17^ Similarly, transition-metal substitution may alter the crystal-field environment of chromophoric ions, thereby affecting electronic transition energies observed in UV-vis spectra.^18,19^ Therefore, combined spectroscopic analysis has significant potential for revealing the relationship between crystal chemistry and colour formation in sugilite aggregates.

In addition to colour genesis, objective colour grading is also important for the gem trade. However, the colour distribution of sugilite aggregates is highly continuous, making traditional visual grading subjective and difficult to reproduce. Conventional clustering methods such as *K*-means require predefined cluster numbers and may inadequately represent transitional colour boundaries in gemstone materials. In contrast, Gaussian Mixture Model (GMM) enables probabilistic characterization of colour distributions,^20^ while Affinity Propagation (AP) can automatically identify cluster structures without predefined class numbers.^21,22^ These methods therefore provide potential advantages for the objective classification of aggregate gemstones with complex colour variation.

The CIE 1976 L*a*b* colour system has been widely applied in gemstone colour evaluation because it enables quantitative characterization of lightness, chroma, and hue.^23–26^ Combined with clustering analysis, this system can provide more objective and reproducible colour classifications than traditional visual grading methods.^27,28^

In this study, 100 sugilite aggregate samples were investigated using colourimetry, FTIR, Raman spectroscopy, UV-vis spectroscopy, XRF, and EPMA. The objectives of this study are: (1) to establish non-destructive spectroscopic indicators for evaluating Al substitution in sugilite aggregates; (2) to clarify the crystal-chemical mechanism responsible for pink colouration; and (3) to develop an objective colour grading framework based on clustering analysis. The results provide new insights into the relationship between crystal chemistry and colour variation in sugilite aggregates and establish a practical non-destructive approach for colour-related compositional evaluation in gemmological materials.

## 2 Experimental

### 2.1 Materials

**Fig. 1:**
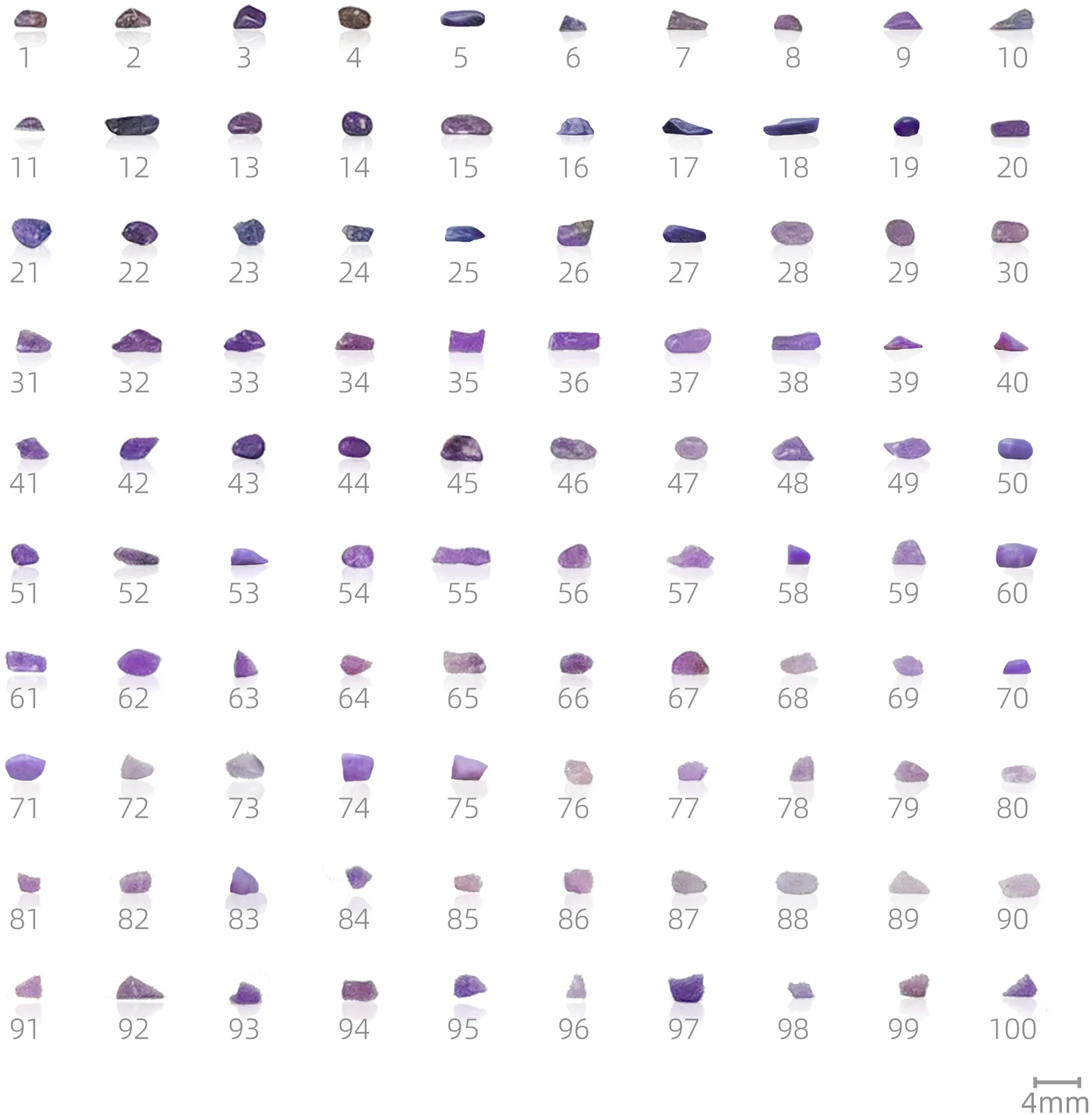
100 sugilite aggregate samples used in this paper.

100 Sugilite aggregate samples were selected, most irregularly shaped and polished with lengths and widths between 2 and 5mm. The thickness of the sample is between 0.5 and 0.7 mm. Fig. 1 shows all the sugilite aggregate samples. Sample numbers are not arranged by colour.

Given that sugilite occurs as polycrystalline aggregates, special attention was paid to sampling representativeness. The selected samples were macroscopically homogeneous in colour, and all analyses were performed on the dominant, uniformly coloured areas to minimise the influence of local mineralogical heterogeneity.

### 2.2 CIE1976 L*a*b* colour system

Colour parameters were quantified using the CIE 1976 L*a*b* colour space, which includes lightness L* and colour coordinates a* and *b*. In the cast-point diagram of a* and b*, the distance from the projection point to the origin of coordinates represents chroma C*, and the angle between the line from the projection point to the origin of coordinates and the +a* axis represents the hue angle *h*°. Chroma C* and hue angle *h*° are derived as follows:1
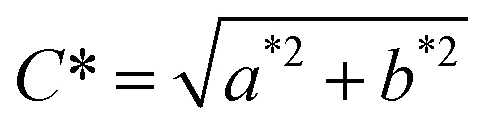
2
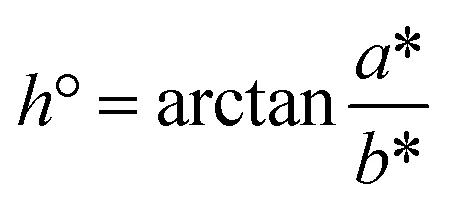


### 2.3 Colour measurement

The colour parameters of sugilite samples were measured using an MDIS-f8 dual integrating sphere spectrometer (Gemmological Experimental Teaching Centre, China University of Geosciences, Beijing), which captures both specular and diffuse reflectance. Measurements were conducted under D65 illumination (CIE 15: 2018) with a spectral range of 380–760 nm at 1 nm resolution. Environmental conditions were maintained at 30 ± 0.5 °C with 220 V AC power (50–60 Hz). The instrument was calibrated using certified reference materials (CRMs) before use.

### 2.4 Spectroscopic analysis

Infrared spectroscopy, Raman spectroscopy, and UV-vis spectroscopy were conducted at the Gem Testing Laboratory, School of Gemmology, China University of Geosciences Beijing (CUGB).

FTIR spectra were collected using a BRUKER Tensor 27 spectrometer (Bremen, Germany) in reflection mode over the range of 400–2000 cm^−1^ with a resolution of 4 cm^−1^ and 8 scans per measurement. The measured area was approximately 1 × 1 mm. Due to the small sample size, both background and sample measurements were performed on a transparent KBr crystal window.

UV-vis absorption spectra were acquired using a GEM-3000 fiber-optic spectrometer (Biaoqi Optoelectronics Technology Development Co., Ltd, Guangzhou, China) over the wavelength range of 220–1000 nm with an integration time of 220 ms, 12 averaged scans, and a boxcar width of 2.

Raman spectra were obtained using a HORIBA HR-Evolution micro-Raman spectrometer (Japan) equipped with a 532 nm laser and a ×50 objective lens. Spectra were collected over the range of 200–4000 cm^−1^ with an integration time of 10 s and laser power of 100 mW. Multiple measurements were performed at different positions on each sample to check for spectral consistency, and the obtained characteristic peak positions were relatively stable, supporting the mineralogical uniformity of the analysed areas. Raman peak assignments were referenced against the RRUFF project database.

### 2.5 Chemical analysis

Chemical analyses by energy-dispersive X-ray fluorescence (EDXRF) were conducted at the Gem Testing Laboratory, School of Gemmology, China University of Geosciences Beijing (CUGB). The chemical compositions of the samples were analyzed using an EDX-7000 energy-dispersive X-ray fluorescence spectrometer. Analyses were performed directly on the polished surfaces of bulk sugilite aggregate samples. Analytical conditions were set to oxide mode with an accelerating voltage of 50 kV, current of 108 µA, dead time of 30%, and a 1 mm collimator. A total of 21 representative samples (3, 9, 10, 11, 12, 13, 36, 42, 46, 52, 56, 62, 65, 70, 76, 78, 81, 83, 86, 97, and 99) were selected from the 100 sugilite aggregate samples for chemical analysis, covering the full colour range of the sample set.

Backscattered electron (BSE) imaging and quantitative wavelength-dispersive electron probe microanalysis (EPMA) were carried out using a Shimadzu EPMA-1720 electron probe microanalyzer at China University of Geosciences Beijing (CUGB), China. Six representative samples (35, 46, 69, 77, 82, and 85) were selected for EPMA analysis. Operating conditions included an accelerating voltage of 15 kV, beam current of 10 nA, beam diameter of 2 µm, and counting times of 10–15 s.

Well-characterized natural and synthetic standards were used for calibration, including diopside (Si, Ca), rutile (Ti), plagioclase (Al), chromite (Cr, Fe), rhodonite (Mn), olivine (Mg), albite (Na), sanidine (K), pentlandite (Ni), vanadium metal (V), celestite (Sr), chalcopyrite (Cu), cobaltite (Co), and sphalerite (Zn). The wavelength-dispersive spectrometry system was equipped with TAP, PET, and LiF analyzing crystals, with a detection limit of approximately 100 ppm. Three to four representative points were analysed in continuous sugilite-rich domains free of visible inclusions, and the average composition was used to minimise the influence of local grain-scale heterogeneity.

### 2.6 Clustering methods

To establish an objective colour grading framework for sugilite aggregates, two unsupervised clustering methods, Gaussian Mixture Model (GMM) and Affinity Propagation (AP), were applied to the CIE 1976 L*a*b* colour data.

GMM is a probabilistic clustering method that assumes the dataset is generated from a mixture of Gaussian distributions with different parameters. Unlike *K*-means clustering, which assigns each sample exclusively to a single cluster, GMM provides probabilistic membership and can better represent gradual colour transitions and heterogeneous colour distributions commonly observed in aggregate gemstones. The optimal number of Gaussian components was determined using the Bayesian Information Criterion (BIC), Akaike Information Criterion (AIC), and silhouette analysis.

AP clustering is an exemplar-based clustering method that does not require the number of clusters to be predefined. Instead, all samples are initially considered as potential cluster centres, and the algorithm iteratively determines the optimal clustering structure through message passing between data points. Compared with conventional partition-based methods, AP clustering is more suitable for datasets with continuous colour distributions and ambiguous cluster boundaries.

The clustering performances of GMM and AP were evaluated and compared using silhouette coefficients, colour coincidence rates, within-group colour differences (Δ*E*00), and the number of suboptimal colour allocations. The clustering results were subsequently used to establish the colour grading framework for sugilite aggregates.

## 3 Results

### 3.1 Colour measurement of sugilite aggregates

**Fig. 2:**
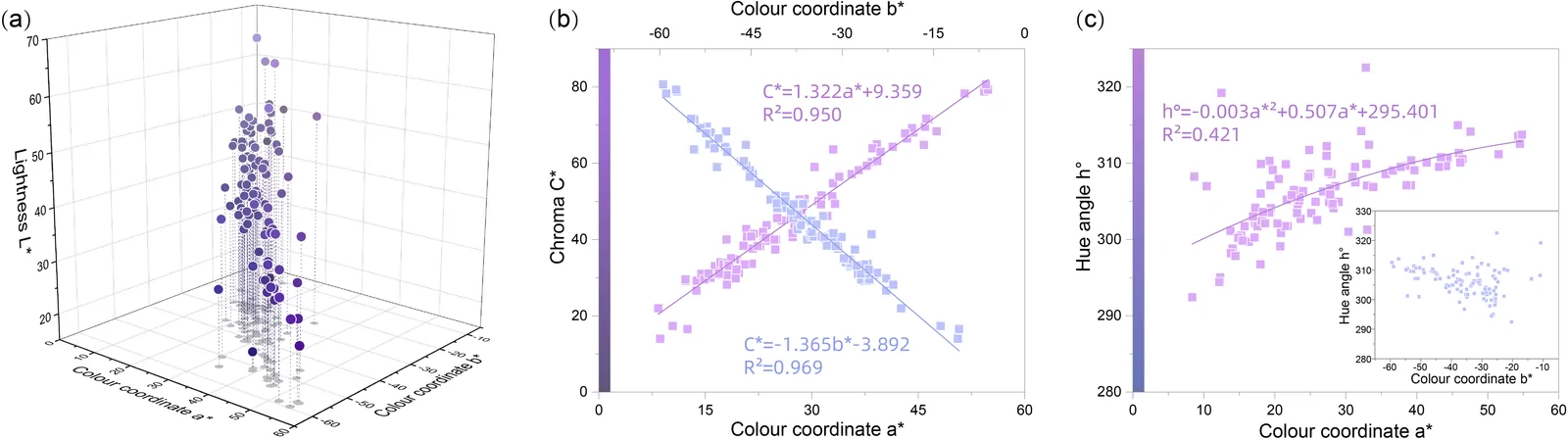
(a) CIE 1976 L*a*b* colour space plot of 100 sugilite aggregate samples. (b) Both a* and b* coordinates exhibit strong correlations with chroma C*, positive for a* (*R*^2^ = 0.950) and negative for b* (*R*^2^ = 0.969). (c) a* is positively correlated with hue angle *h*°, whereas b* shows a weaker negative correlation (*R*^2^ = 0.186).

Colour parameters of the 100 sugilite aggregate samples under the D65 light source are as follows: L* (18.52, 68.56), a* (8.38, 54.82), b* (−59.43, −10.79), chroma C* (13.99, 80.67), hue angle *h*° (292.43, 322.53). Fig. 2(a) shows the three-dimensional projection plot of the 100 samples' colour parameters.

Colour analysis of 100 samples revealed significant linear relationships between chromatic parameters. As shown in Fig. 2(b), both a* and b* were strongly correlated with chroma C*, but in opposite directions: a* showed a positive correlation (Pearson's *r* = 0.975, *R*^2^ = 0.950), whereas b* exhibited a negative correlation (*r* = −0.984, *R*^2^ = 0.969). Thus, samples with higher chroma generally exhibit simultaneously enhanced red (+a*) and blue (−b*) components, consistent with the characteristic purple colour of sugilite aggregates.

As shown in Fig. 2(c), *h*° increases with increasing a* (*r* = 0.642, *R*^2^ = 0.421) and tends to decrease with increasing b* (*r* = −0.323, *R*^2^ = 0.186). Because all samples are located in the fourth quadrant of the CIELAB colour space, their hue is determined by the relative contributions of the positive a* (red) and negative b* (blue) components rather than by either coordinate independently. Therefore, the transition from purple toward pink-purple is better represented by the change in their relative balance, expressed by *h*°, with increasing *h*° corresponding to a relatively stronger red contribution and/or weaker blue contribution.

### 3.2 GMM and AP clustering of sugilite aggregates

To determine the optimal number of Gaussian components, the Bayesian Information Criterion (BIC), Akaike Information Criterion (AIC), and the silhouette coefficient were jointly evaluated (Fig. 4(a) and (b)).

As shown in Fig. 4(a), the BIC remains nearly constant at approximately 650 for *k* = 2 to 4, indicating that models with 2 to 4 components achieve a statistically equivalent goodness-of-fit.^29^ From *k* = 5 onward, the BIC generally increases, reaching near 750 at *k* = 10. The AIC decreases sharply from *k* = 2 (≈600) to *k* = 3 (≈560), with only marginal improvement thereafter (≈550 at *k* = 4).

The silhouette coefficient (Fig. 4(b)) provides critical validation. It decreases from ≈0.5 at *k* = 2 to ≈0.27 at *k* = 3, then to ≈0.2 at *k* = 4, and progressively approaches 0.0 at *k* = 6. Given that a silhouette score below 0.25 generally indicates negligible cluster structure, *k* = 3 represents the last reasonable configuration.

Considering the BIC equivalence among *k* = 2–4, the dominant AIC improvement at *k* = 3, and the rapid deterioration of silhouette scores beyond *k* = 3, the model with three Gaussian components was selected as optimal.

Affinity Propagation (AP) clustering does not require a predefined number of clusters; instead, it automatically determines the number based on the data. The method primarily relies on two parameters: preference and damping factor. The preference value determines the likelihood of a sample becoming a cluster centre and serves as the key parameter for adjusting the number of clusters. A higher *p* value results in more cluster centres, with the extreme case being each sample as its own centre. Typically, this value is initialized as the median or mean of the similarity matrix. The damping factor mitigates numerical oscillations during iterations (default: 0.5) and has a minor impact on clustering results. A higher damping factor (closer to 1) slows convergence but enhances stability, while a lower value (closer to 0.5) speeds up convergence but may reduce stability. To select the optimal preference value for this dataset, a systematic search was performed over a range of preference from −20 to 0. For each candidate preference, the resulting number of clusters and the corresponding silhouette coefficient were evaluated (Fig. 4(c) and (d)).

As shown in Fig. 4(c), the silhouette coefficient varies with preference, reaching its maximum at preference = −12.65. At this optimal preference, the automatically determined number of clusters is 4 (Fig. 4(d)). Preference values higher than −12.65 produce more than 4 clusters, while lower values result in lower silhouette scores.

Consequently, AP clustering with the optimal preference = −12.65 produced 4 distinct groups from the 100 sugilite aggregates.

**Fig. 3:**
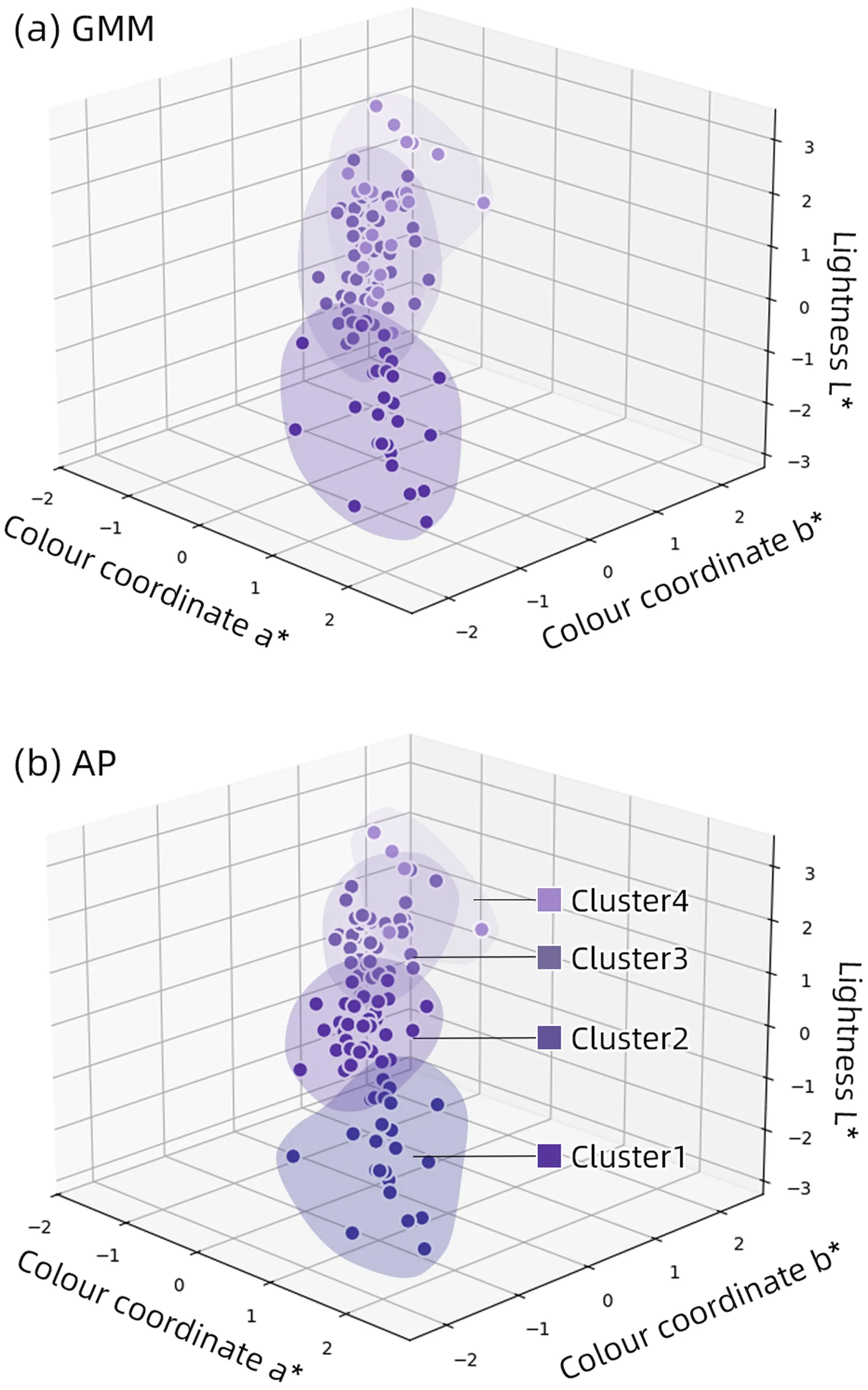
(a) The three-dimensional scatter plot of GMM; (b) the three-dimensional scatter plot of AP.

The clustering results of the 100 sugilite aggregates using GMM and AP methods yielded 3 and 4 distinct groups, respectively (Fig. 3(a) and (b)).

**Fig. 4:**
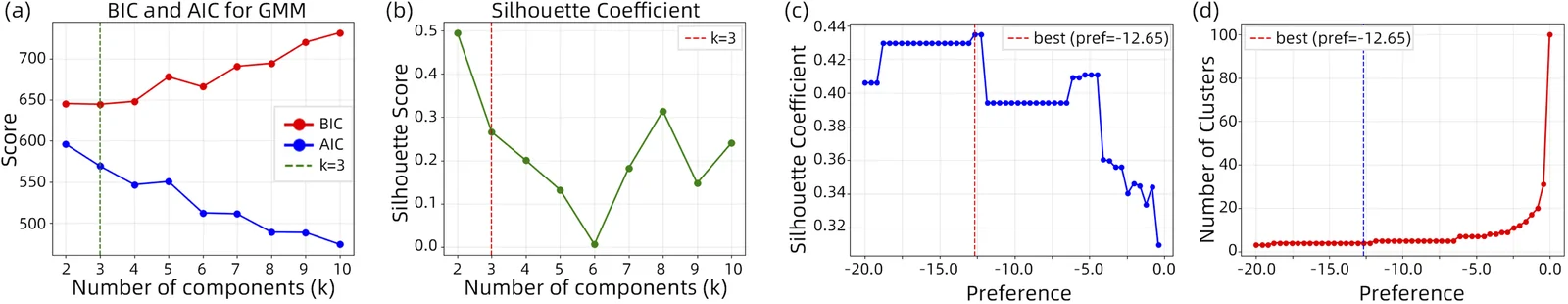
Optimization of clustering algorithms. Gaussian Mixture Model (GMM): (a) BIC/AIC *vs.* number of components, (b) silhouette coefficient *vs.* number of components. Affinity Propagation (AP) clustering: (c) silhouette coefficient *vs.* preference, (d) number of clusters *vs.* preference. Optimal parameters are indicated by dashed lines (red for silhouette coefficient, blue for number of clusters, green for BIC/AIC).

### 3.3 Implications of clustering analysis for sugilite aggregate colour grading

The clustering performance of GMM and AP was compared using the coincidence rate, mean within-group Δ*E*00, and the number of suboptimal Δ*E*00 allocations (Table 1). GMM partitioned the data into three clusters, with coincidence rates ranging from 66.00% to 86.21% and mean within-group Δ*E*00 values from 4.87 to 6.43. A total of 28 out of 100 samples were identified as suboptimal Δ*E*00 allocations. In comparison, AP automatically identified four clusters, with coincidence rates of 79.17–100.00%, lower mean within-group Δ*E*00 values of 3.49–4.92, and only nine suboptimal allocations. Thus, for the present dataset and colour-based evaluation criteria, the four-cluster AP solution showed greater within-group colour homogeneity than the three-cluster GMM solution.

The cluster results for sugilite aggregates of GMM and AP

**Table:** 

Methods		Cluster centre		
GMM		1	2	3
	Simulated colour	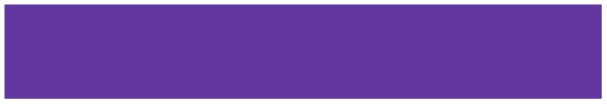	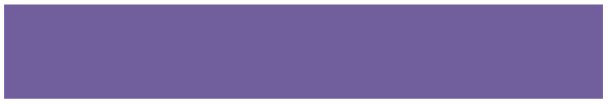	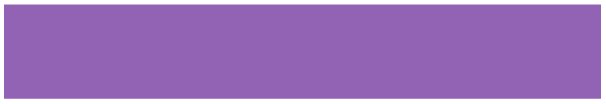
	L*	33.90	43.99	49.75
	a*	41.44	21.79	34.42
	b*	−49.87	−31.86	−35.84
	Coincidence rate	86.21%	66.00%	66.66%
	Mean Δ*E*00 within the group	4.87	5.53	6.43
	Suboptimal Δ*E* allocations	28/100		
	Member	29	50	21

**Table:** 

AP		1	2	3	4
	Simulated colour	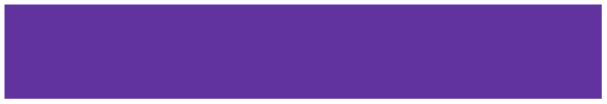	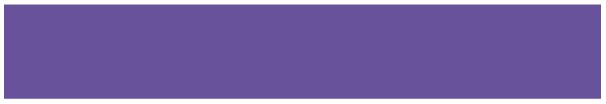	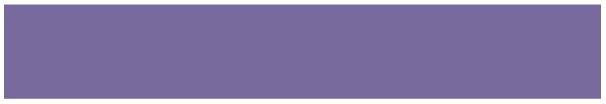	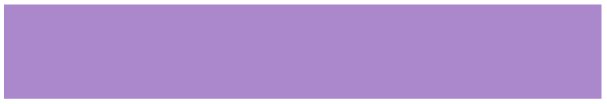
	L*	32.61	40.32	48.10	61.87
	a*	43.14	26.46	17.00	26.88
	b*	−50.84	−37.19	−25.49	−30.41
	Coincidence rate	79.17%	94.44%	94.29%	100.00%
	Mean Δ*E*00 within the group	4.30	3.49	4.28	4.92
	Suboptimal Δ*E* allocations	9/100			
	Member	24	36	35	5

The three-cluster GMM and four-cluster AP solutions can be regarded as different levels of partitioning of the same continuous colour distribution rather than contradictory classifications. The GMM solution provides a broader three-group partition, whereas AP further separates a small, light-coloured group (cluster 4; mean L* = 61.87) at the light end of the colour continuum. This additional distinction provides a finer resolution for practical colour grading.

Based on the greater within-group colour homogeneity of the AP solution and its ability to distinguish this light-coloured group, the four-cluster AP model was adopted as the basis for the proposed colour grading scheme. In combination with the Bellerophon Gemlab colour terminology, four colour grades were defined for sugilite aggregates.^30^ Cluster 1 (lowest brightness, highest saturation): deep purple; cluster 2 (second lowest brightness, second highest saturation): vivid purple; cluster 3 (lowest saturation): purple; cluster 4 (lightest colour): pastel purple (Fig. 3(b)).

### 3.4 FTIR and Raman spectroscopy

Sugilite belongs to the osumilite–milarite group and is structurally characterized by a [Si_12_O_30_] double-ring silicate framework.^1^ The infrared and Raman spectra of the studied samples consistently display the characteristic vibrational features of sugilite and reveal systematic spectral variations related to compositional differences.

**Fig. 5:**
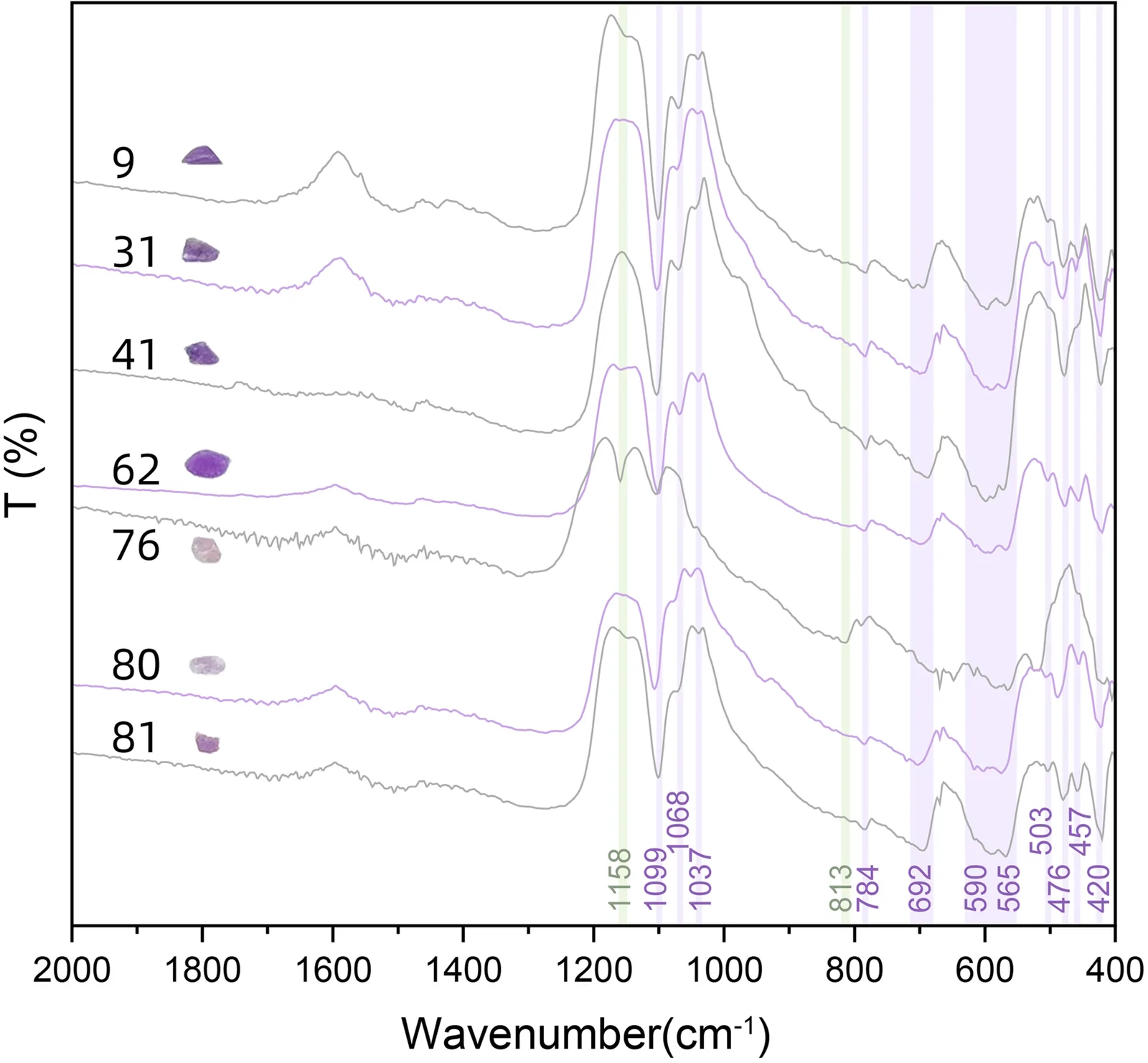
(a) Raman spectra of sample 3, 11, 46, 77, and 86. (b) Related FTIR spectra of samples.

The FTIR spectra of all samples exhibit characteristic absorption bands associated with the sugilite structure (Fig. 5). The principal vibrational modes of the silicate framework include terminal Si–O and bridging Si–O–Si vibrations. Characteristic bands at approximately 1099, 1068, and 1037 cm^−1^ are assigned to *ν*_as_Si–O–Si, *ν*_as_O–Si–O, and *ν*_s_O–Si–O vibrations, respectively.^6,31^ Bands within the 784–565 cm^−1^ region correspond mainly to *ν*_a_Si–O–Si vibrations, whereas those in the 503–420 cm^−1^ region are related to *δ*Si–O and M–O vibrations.^6,32^ The distribution of the principal FTIR characteristic peaks is summarized in Table 2.

**Table 2:** Distribution of FTIR characteristic peak positions of sugilite

Characteristic peak	The vibrational mode
1099 cm^−1^	*ν*_as_Si–O–Si
1068 cm^−1^	*ν*_as_O–Si–O
1037 cm^−1^	*ν*_s_O–Si–O
784–565 cm^−1^	*ν*_s_Si–O–Si
503–420 cm^−1^	*δ*Si–O and M–O

In several lighter-coloured samples (*e.g.*, samples 76 and 81), additional quartz-related absorption bands were identified, including Si–O asymmetric stretching vibrations near 1158 cm^−1^ and Si–O–Si stretching vibrations near 813 cm^−1^. Minor pectolite-related features were occasionally observed within the 800–1050 cm^−1^ region. Nevertheless, FTIR spectroscopy confirms that sugilite is the dominant mineral phase in all investigated samples.

**Fig. 6:**
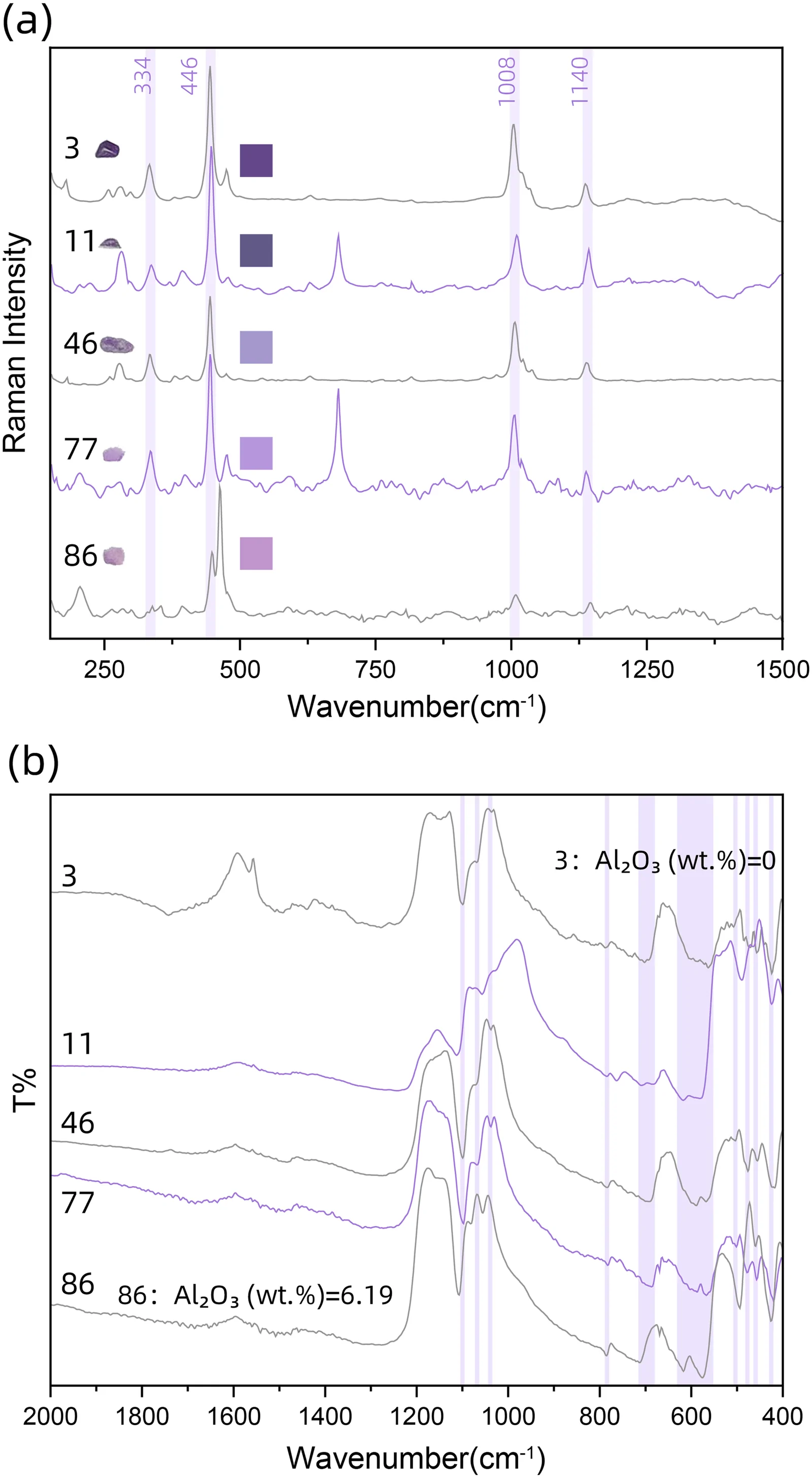
The FTIR spectra of sugilite aggregates. The characteristic infrared peaks of sugilite are marked in purple, while those of quartz are indicated in green.

Raman spectroscopy further supports the mineral identification results. Comparison with the RRUFF database indicates that the principal Raman peaks of sugilite occur at approximately 334, 446, 1008, and 1139 cm^−1^ (Fig. 6(a)). The band near 334 cm^−1^ is assigned to *δ*Si–O–Si bending vibrations,^33^ whereas the peak around 446 cm^−1^ corresponds to breathing or symmetric bending vibrations of the hexagonal double-ring silicate structure.^34,35^ Peaks within the 950–1100 cm^−1^ region are attributed to Si–O stretching vibrations.^36^ In addition to sugilite, Raman spectra also identified minor associated minerals, including richterite in samples 11 and 77 and quartz in samples 77 and 86. Nevertheless, the characteristic vibrational features of sugilite remain dominant in the analysed spectra, indicating that these minerals occur as subordinate associated phases.

A comparison of the FTIR and Raman spectra reveals consistent positional variations of the characteristic peaks among different samples. As shown in Table 3, Fig. 6(a) and (b), samples exhibiting higher Raman peak positions also display corresponding shifts of FTIR characteristic troughs toward higher wavenumbers. For example, the principal Raman peaks of sample 86 are approximately 3–10 cm^−1^ higher than those of sample 3, and similar trends are observed in the infrared spectra. These systematic spectral shifts suggest that the local crystal-chemical environment of sugilite varies among samples and may be associated with compositional substitution within the crystal structure.

**Table 3:** Specific wavenumbers of Raman and IR characteristic peaks (cm^−1^)

	3	11	46	77	86
Raman	333	337	335	335	338
	445	447	445	445	448
	1005	1010	1007	1007	1008
	1136	1143	1140	1138	1146
FTIR	424	422	419	420	426
	457	463	455	457	461
	471	490	476	478	496
	563	579	567	567	575
	692	683	689	687	714
	787	764	783	783	785
	1036	1057	1038	1040	1057
	1069	1078	1069	1069	1082
	1099	1113	1099	1099	1107

The consistent variations observed in both FTIR and Raman spectra indicate that vibrational spectroscopy is sensitive to structural and compositional changes in sugilite. These spectral characteristics provide the basis for the subsequent discussion of Al substitution and its relationship with colour variation.

### 3.5 UV-vis spectroscopy

**Fig. 7:**
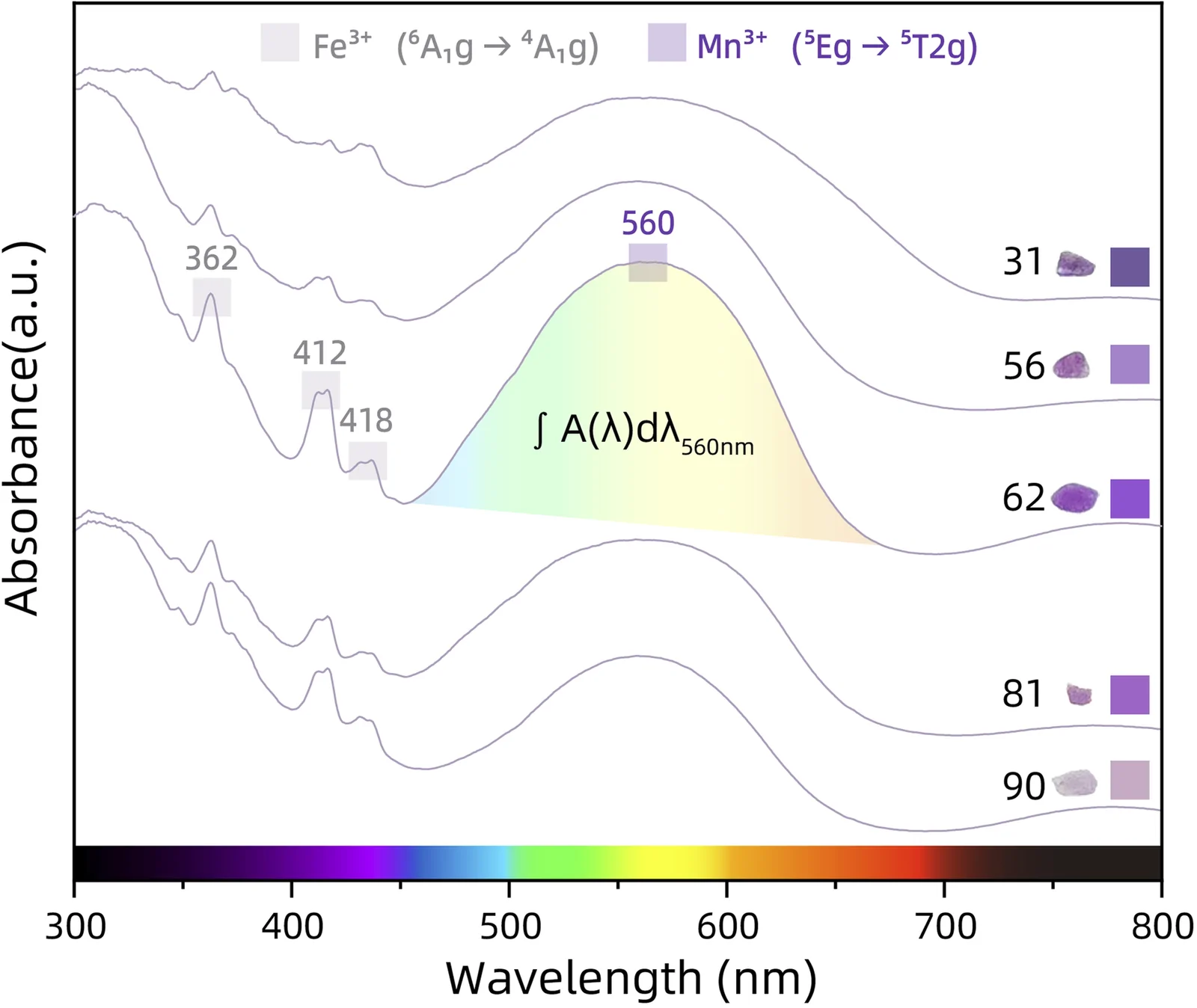
The UV-vis spectra conform to the absorption of Mn^3+^ in sugilite aggregates.

As shown in Fig. 7, samples 31, 56, 62, 81, and 90 exhibit a broad absorption band centred at 540–580 nm, consistent with Mn^3+^-related absorptions reported in other purple minerals.^37–40^ This band is attributed to the spin-allowed ^5^E_g_ → ^5^T_2g_ transition of Mn^3+^, associated with Jahn–Teller splitting of the ^5^T_2g_ level.^41^ The absorption occurs mainly within the yellow–green region of the visible spectrum.

In addition, several relatively weak and narrow absorption peaks are observed between 300 and 500 nm. Previous studies have demonstrated that iron in sugilite occurs predominantly as Fe^3+^ occupying octahedrally coordinated A sites.^1,13^ The peaks at 362, 412, and 418 nm are therefore assigned to Fe^3+^ electronic transitions (^6^A_1g_ → ^4^A_1g_, ^4^E_g_ (^4^G)).^42,43^ Although similar absorptions have also been reported for Mn^2+^,^42^ electron paramagnetic resonance (EPR) studies confirmed the absence of Mn^2+^ in sugilite,^44,45^ supporting the assignment to Fe^3+^.

### 3.6 ED-XRF

Quantitative ED-XRF analyses were performed on 21 selected sugilite aggregate samples. Most samples exhibit relatively homogeneous surface colouration despite minor local heterogeneity. The major chemical components are SiO_2_, Fe_2_O_3_, K_2_O, Mn_2_O_3_, and Al_2_O_3_. The compositional ranges (wt%) are: SiO_2_ = 65.25–96.82, Fe_2_O_3_ = 0.603–19.045, K_2_O = 0.81–6.187, Mn_2_O_3_ = 0.106–2.707, Al_2_O_3_ = 0.000–6.698, and MgO = 0.000–16.329. Minor components include CaO, SrO, Cr_2_O_3_, V_2_O_5_, TiO_2_, ZnO, and CuO.

Among the transition-metal elements, Mn and Fe are present in all purple samples, consistent with previous studies.^5,10,11,44^ Samples 52, 81, and 83 show unusually high SiO_2_ contents, corresponding to the presence of associated quartz identified by infrared spectroscopy. Several samples also exhibit elevated MgO contents; for example, sample 11 contains 16.329 wt% MgO, which is attributed to associated richterite identified by Raman and infrared analyses. In contrast, Cr_2_O_3_, TiO_2_, ZnO, and CuO are below detection limits in most samples.

### 3.7 BSE and EPMA

**Fig. 8:**
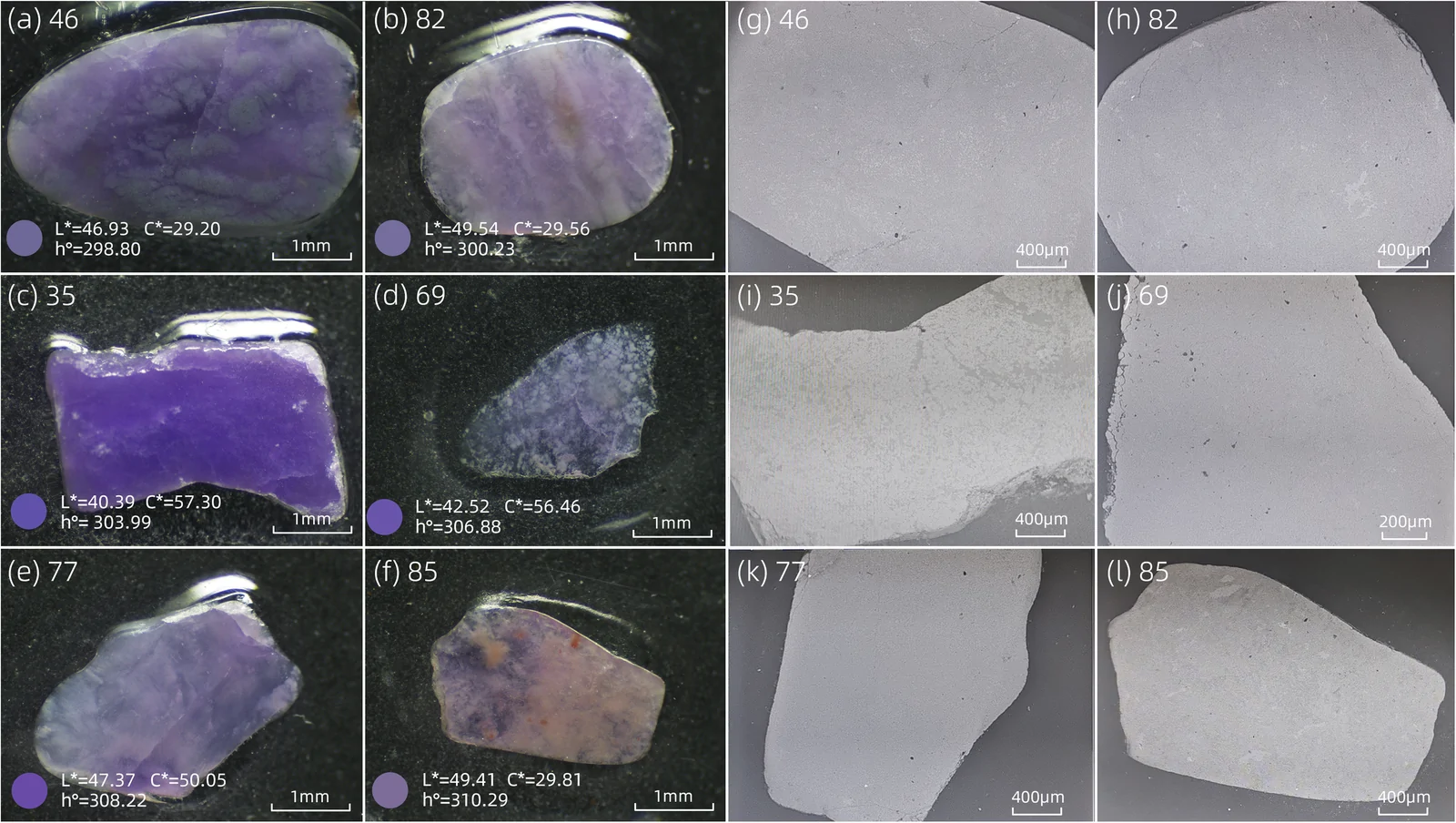
(a–f) Photomicrographs and colour data of samples 46–85; (g–l) BSE images of samples 46–85, all of which are predominantly composed of sugilite. Sample 46 contains minor amounts of pectolite (light grey patches and acicular minerals) and aegirine (light-coloured spotty minerals). Sample 82 contains minor aegirine. Sample 35 contains minor quartz (dark grey spotty minerals). Sample 69 contains minor amphibole. Sample 85 contains minor pectolite (light grey patchy and acicular minerals).

Because sugilite occurs as polymineralic aggregates, six representative samples were selected for BSE imaging and EPMA analyses to characterize mineral assemblages and compositional variations (Fig. 8). As shown in Fig. 8(a)–(f), the hue angle (*h*°) of samples gradually increases, corresponding to a colour transition from purple to pinkish purple.

BSE images reveal relatively homogeneous phase distributions with needle-like, granular, and blocky textures (Fig. 8(g–l)). SEM-EDS analyses indicate that all six samples are composed predominantly of sugilite, accompanied by minor amounts of pectolite, aegirine, and quartz.

In BSE images, the sugilite matrix appears medium grey. Pectolite occurs as light-grey point-like, needle-like, and patchy domains within the sugilite matrix (Fig. 8(g, j and l)). Aegirine appears as bright point-like minerals with relatively high b ackscattered electron intensity (Fig. 8(g, h and j–l)), whereas quartz occurs as dark-grey granular domains (Fig. 8(i)). The observed mineral intergrowth textures are consistent with metasomatic replacement features.

For each sample, three to four representative points were selected for EPMA analyses, and the averaged chemical compositions are listed in Table 4.

**Table 4:** EPMA analysis of sugilite from six samples, showing average chemical compositions (with 3–4 measurement points per sample, values in wt%). Li was not detected, so the reported totals are below 100%

Component (wt%)	35	46	69	77	82	85
SiO_2_	71.36	70.98	70.84	71.69	71.22	72.64
Fe_2_O_3_	12.46	13.49	10.47	9.55	13.19	6.00
Na_2_O	6.11	6.11	6.06	6.22	6.07	6.12
K_2_O	4.75	4.68	4.64	4.71	4.68	4.80
Al_2_O_3_	0.74	0.28	3.48	3.76	0.64	6.13
Mn_2_O_3_	0.61	0.25	0.26	0.11	0.10	0.13
CuO	0.05	0.00	0.04	0.01	0.06	0.03
MgO	0.05	0.01	0.08	0.02	0.03	0.01
BaO	0.01	0.00	0.05	0.01	0.00	0.01
V_2_O_5_	0.01	0.01	0.01	0.00	0.01	0.01
SO_3_	0.00	0.01	0.00	0.00	0.00	0.01
CaO	0.00	0.04	0.00	0.00	0.00	0.00
P_2_O_5_	0.00	0.02	0.02	0.00	0.00	0.03
Co Kα	0.00	0.00	0.00	0.03	0.00	0.00
Total	96.15	95.87	95.96	96.11	96.01	95.92

## 4 Discussion

### 4.1 Nondestructive spectroscopic assessment of Al abundance in sugilite

**Fig. 9:**
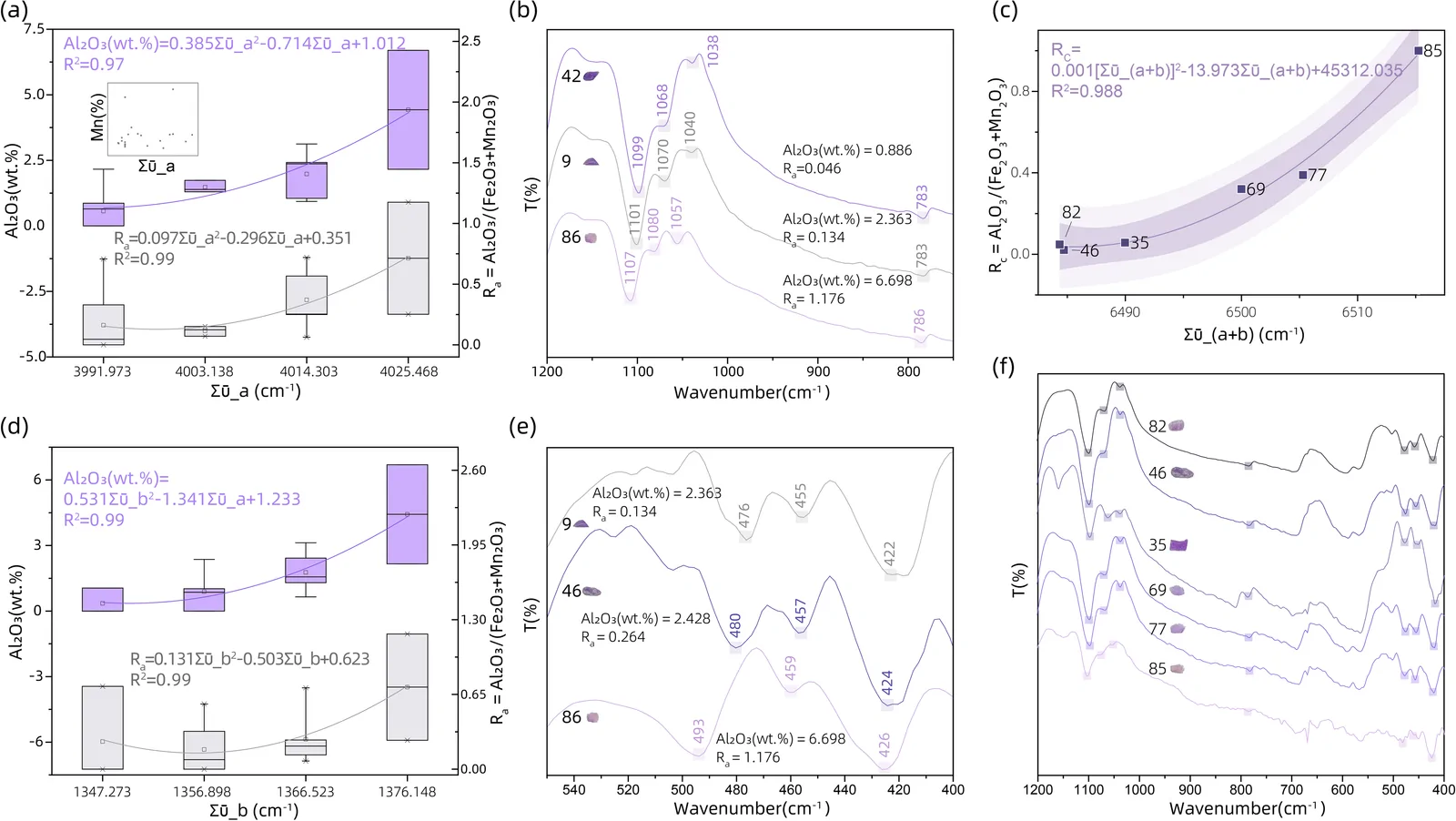
(a) Relationships of Σ*ῡ*_*a* (sum of the four characteristic trough wavenumbers in the 1150–700 cm^−1^ region) with Al_2_O_3_ content (wt%) and *R*_a_ = Al_2_O_3_/(Fe_2_O_3_ + Mn_2_O_3_) derived from XRF data, showing positive correlations. (b) FTIR spectra of samples 42, 9, and 86. With increasing Al_2_O_3_ content and *R*_a_ values, the four characteristic troughs in the 1150–700 cm^−1^ region progressively shift toward higher wavenumbers. (c) Relationship between Σ*ῡ*_(*a* + *b*) (sum of characteristic trough wavenumbers in the 1150–700 and 503–420 cm^−1^ regions) and the oxide mass ratio *R*_c_ = Al_2_O_3_/(Fe_2_O_3_ + Mn_2_O_3_) calculated from EPMA data of sugilite, showing a positive correlation. (d) Relationships of Σ*ῡ*_*b* (sum of the three characteristic trough wavenumbers in the 503–420 cm^−1^ region) with Al_2_O_3_ content (wt%) and *R*_a_ derived from XRF data, showing positive correlations. (e) FTIR spectra of samples 9, 46, and 86. The three characteristic troughs in the 503–420 cm^−1^ region progressively shift toward higher wavenumbers with increasing Al_2_O_3_ content and *R*_a_ values. (f) FTIR spectra of samples selected for EPMA analyses. From top to bottom, the characteristic troughs in both the 1150–700 and 503–420 cm^−1^ regions shift toward higher wavenumbers.

Systematic shifts were observed in both the infrared and Raman spectra of the studied sugilite samples. In the infrared spectra (Fig. 9(b) and (e)), the characteristic transmission troughs within the ranges of 1150–700 cm^−1^ and 503–420 cm^−1^ exhibited progressive positional variations among different samples. Correspondingly, the Raman peaks at 334, 446, 1008, and 1139 cm^−1^ also showed noticeable shifts (Fig. 6(a) and (b)). These spectral variations indicate changes in the local crystal chemical environment associated with compositional differences among samples.

Sugilite has the general chemical formula KNa_2_(Fe,Mn,Al)_2_Li_3_Si_12_O_30_ and commonly undergoes isomorphic substitution, particularly among Fe, Mn, and Al at the A site of the lattice.^1,12^ In silicate minerals, Al^3+^ may enter the structure either through tetrahedral coordination by replacing Si^4+^ or through octahedral coordination by occupying M sites.^46,47^ If Al^3+^ substitutes for Si^4+^ in tetrahedral sites, the longer and weaker Al–O bond relative to the Si–O bond would reduce the vibrational frequency of the Si–O–Si framework, leading to a redshift in the infrared spectrum. However, the experimental results revealed the opposite trend.

Fig. 9(a) shows the relationships between the sum of the wavenumbers of the four characteristic transmission troughs within the 1150–700 cm^−1^ region (denoted as Σ*ῡ*_*a*) and both the Al content and the Al_2_O_3_/(Fe_2_O_3_ + Mn_2_O_3_) ratio obtained by XRF for 21 sugilite aggregate samples (3, 9, 10, 11, 12, 13, 36, 42, 46, 52, 56, 62, 65, 70, 76, 78, 81, 83, 86, 97, and 99). Strong positive correlations were observed between Σ*ῡ*_*a* and Al content (*R*^2^ = 0.97) as well as between Σ*ῡ*_*a* and the Al_2_O_3_/(Fe_2_O_3_ + Mn_2_O_3_) ratio (*R*^2^ = 0.97), whereas no significant correlation was identified with Mn content. Representative spectra from samples 42, 9, and 86 (Fig. 9(b)) further demonstrate that increasing Al content corresponds to systematic shifts of the characteristic troughs toward higher wavenumbers.

This behaviour suggests that Al^3+^ does not predominantly replace Si^4+^ in tetrahedral sites, but instead mainly occupies octahedral M sites. Because the ionic radius of Al^3+^ (0.535 Å) is smaller than those of Fe^3+^ and Mn^3+^ (0.645 Å), substitution by Al^3+^ contracts the octahedral units and increases the structural tension of the adjacent [Si_12_O_30_] silicate framework. This structural contraction strengthens the Si–O bonds and increases the corresponding force constants, resulting in systematic blueshifts of vibration modes involving Si–O stretching.

The transmission troughs within the 503–420 cm^−1^ region displayed a similar trend. Fig. 9(d) illustrates the relationships between Σ*ῡ*_*b*, defined as the sum of the wavenumbers of the three characteristic troughs in this region, and both Al content and the Al_2_O_3_/(Fe_2_O_3_ + Mn_2_O_3_) ratio. Positive correlations were again observed, indicating that substitution of Fe–O and Mn–O bonds by Al–O bonds increases the overall vibrational frequency of the silicate framework. As shown in Fig. 9(e), samples 9, 46, and 86 exhibit progressive shifts toward higher wavenumbers with increasing Al_2_O_3_ content.

The infrared results are further supported by Raman spectroscopy. As described in Section 3.4 and shown in Fig. 6(a), (b) and Table 3, the principal Raman peaks of sugilite aggregates also exhibit systematic positional variations. For example, the Raman peak positions of sample 86 are 3–10 cm^−1^ higher than those of sample 3, corresponding to a difference of 6.19 wt% in Al_2_O_3_ content. The consistency between the infrared and Raman spectral shifts confirms that the observed spectral variations are primarily associated with Al substitution at the A site.

Although XRF measurements may be influenced by associated minerals and non-structural Al, the synchronous shifts of both Σ*ῡ*_*a* and Σ*ῡ*_*b* strongly suggest that the spectral variations are dominantly controlled by Al substitution within the sugilite structure. To further verify this interpretation, EPMA and SEM analyses were conducted on six representative samples. SEM observations indicate that all six thin sections are dominated by sugilite as the principal mineral phase (Fig. 8(g–l)), minimizing interference from associated minerals.

Table 5 summarizes the relationships among hue angle (*h*°), Σ*ῡ*_(*a* + *b*) (the combined sum of characteristic trough wavenumbers from both infrared regions), Σ*λ* (the sum of the starting, maximum, and ending wavelengths of the main absorption band near 560 nm), and the Al_2_O_3_, Fe_2_O_3_, and Mn_2_O_3_ contents measured by EPMA. Σ*ῡ*_(*a* + *b*) exhibits a strong positive correlation with Al_2_O_3_ content (*r* = 0.987) and the Al_2_O_3_/(Fe_2_O_3_ + Mn_2_O_3_) ratio (*r* = 0.945), together with a strong negative correlation with Fe_2_O_3_ content (*r* = −0.985).

**Table 5:** Pearson correlation coefficients (*r*) and significance levels (Sig.) among hue angle (*h*°), infrared spectral parameters, UV-vis spectral parameters, and chemical compositions (EPMA data) of six representative sugilite aggregate samples. Definitions of the summation parameters used in this table: Σ*ῡ*_(*a* + *b*) = the sum of characteristic trough wavenumbers in the 1150–700 cm^−1^ and 503–420 cm^−1^ FTIR regions. Σ*λ* = the sum of the starting wavelength, peak maximum wavelength, and ending wavelength of the main Mn^3+^ absorption band centred at ∼560 nm in the UV-vis spectra. This parameter describes the overall spectral position of the absorption band. **Correlation is significant at the 0.01 level (two-tailed). *Correlation is significant at the 0.05 level (two-tailed)

		*h*°	Σ*ῡ*_(*a* + *b*)	Σ*λ*	Al_2_O_3_	Fe_2_O_3_	Mn_2_O_3_	Al_2_O_3_/(Fe_2_O_3_ + Mn_2_O_3_)
*h*°	*r*	1	0.962**	−0.904*	0.932**	−0.922**	−0.168	0.848*
	Sig.		0.002	0.013	0.007	0.009	0.751	0.033
Σ*ῡ*_(*a* + *b*)	*r*	0.962**	1	−0.817*	0.987**	−0.985**	−0.334	0.945**
	Sig.	0.002		0.047	0	0	0.518	0.005
Σ*λ*	*r*	−0.904*	−0.817*	1	−0.786	0.8	0.137	−0.725
	Sig.	0.013	0.047		0.064	0.056	0.795	0.103

Fig. 9(c) further demonstrates a strong positive correlation (*R*^2^ = 0.988) between Σ*ῡ*_(*a* + *b*) and the measured chemical compositions of the six samples. The systematic shifts of the infrared characteristic troughs among these samples are shown in Fig. 9(d). These results demonstrate that the infrared spectral parameters, particularly Σ*ῡ*_(*a* + *b*), can serve as non-destructive spectroscopic indicators for screening Al substitution and relative Al abundance in sugilite. Within the compositional range investigated in this study, systematic FTIR shifts were observed with increasing Al content. However, because these shifts represent a continuous response to compositional substitution rather than the appearance of a discrete Al-specific band, a strict Al-content detection threshold cannot be defined from the present dataset. Establishing such a threshold would require a larger set of compositionally homogeneous reference samples and repeated spectroscopic measurements to quantify analytical reproducibility and prediction uncertainty. Raman spectroscopy showed consistent compositional sensitivity but is currently considered complementary rather than independently quantitative because of the more limited dataset.

### 4.2 The pink colour genesis of sugilite aggregates

**Fig. 10:**
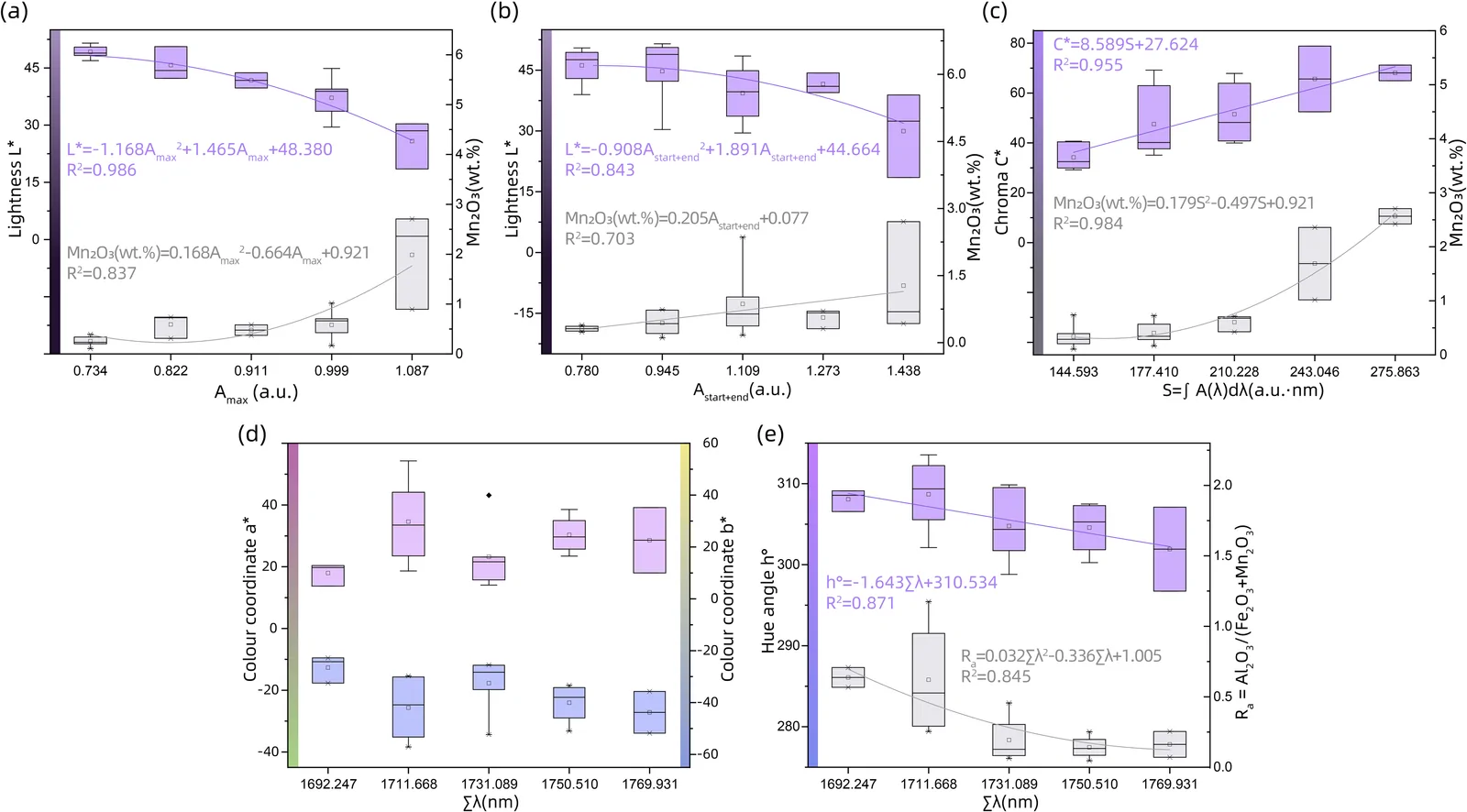
(a) Maximum absorbance *A*_max_ of the main absorption band at ∼560 nm *versus* lightness L* and XRF-derived Mn_2_O_3_ (wt%); (b) baseline absorption (*A*_start+end_) versus L* and Mn_2_O_3_ (wt%); (c) integrated area of the main absorption band [*S* = ∫*A*(*λ*)d*λ*] *versus* chroma C* and Mn_2_O_3_ (wt%); (d) distributions of the CIELAB a* and b* coordinates at different values of the overall spectral–position parameter Σ*λ*; (e) overall spectral position parameter Σ*λ versus* hue angle *h*° and the XRF-derived ratio *R*_a_ = Al_2_O_3_/(Fe_2_O_3_ + Mn_2_O_3_).

Fig. 10(a) and (b) illustrate the relationships between the maximum intensity of the main absorption band at approximately 560 nm (*A*_max_), the baseline absorption (*A*_start+end_), lightness (L*), and Mn content (wt%) obtained by XRF analysis for sugilite aggregates. The parameter *A*_start+end_, defined as the sum of the absorbance values at the beginning and the end of the main absorption band, was used to evaluate the overall background absorption level in the UV-vis diffuse reflectance spectra. The starting and ending wavelengths were determined from the local minima bounding the principal Mn^3+^ absorption band.

The results show that *A*_max_ exhibits a strong negative correlation with L* (*R*^2^ = 0.986), indicating that samples with stronger absorption bands display darker colours and lower lightness. In addition, *A*_max_ is positively correlated with Mn content (*R*^2^ = 0.837), confirming that Mn is the principal chromophore responsible for the pink-purple colouration of sugilite aggregates. Similarly, *A*_start+end_ also shows correlations with both L* (*R*^2^ = 0.843) and Mn content (*R*^2^ = 0.703), suggesting that increasing Mn concentration not only enhances the intrinsic absorption around 560 nm, but also increases the background absorption by deepening the overall colour and strengthening light-scattering effects.

In UV-vis diffuse reflectance spectroscopy, the measured “absorption” is actually an apparent absorption influenced by both intrinsic absorption and scattering effects. On one hand, the increase in *A*_max_ with decreasing L* reflects stronger intrinsic absorption associated with Mn^3+^ electronic transitions near 560 nm, indicating a higher concentration of chromophoric ions. On the other hand, the lightening of colour in aggregate gemstones cannot always be attributed solely to a decrease in chromophore concentration. In some samples, reduced Mn content weakens the intrinsic absorption and results in higher reflectance and lower baseline absorption. In other samples, however, enhanced scattering caused by fissures, microcrystalline textures, or greater sample thickness increases the apparent absorption, leading to relatively high baseline absorption despite lighter colours. Therefore, the colour depth of sugilite aggregates is controlled not only by Mn concentration, but also by aggregate texture and scattering effects.

Fig. 10(c) shows the relationship between chroma C* and the integrated area of the main absorption band, expressed as *S* = ∫*A*(*λ*)d*λ*. A significant positive correlation is observed, indicating that the overall absorption intensity of the 560 nm band is the primary factor controlling chroma. Furthermore, the absorption area also shows a positive correlation with Mn content, demonstrating that increasing Mn concentration not only darkens the colour, but also enhances colour saturation.

Minor associated minerals may also exert secondary effects on the bulk colour coordinates of the sugilite aggregates. Light-coloured or colourless phases such as quartz and pectolite may dilute the colour of the sugilite matrix, potentially increasing L* and reducing chroma, whereas Fe-rich phases such as aegirine may decrease lightness and modify the overall colour appearance. Richterite occurs only as a minor associated phase and is therefore unlikely to be a primary control on the systematic variations in a* and b*. Although such mineralogical heterogeneity cannot be completely excluded from bulk colour and XRF measurements, the predominance of sugilite in the investigated samples, together with the systematic relationships between colour parameters, sugilite composition, and spectroscopic features, indicates that the principal colour variation is controlled by the chemistry of the sugilite phase itself, particularly Mn-related colouration and Al–Fe–Mn substitution.

To further examine how the spectral position of the principal Mn^3+^ absorption band is related to the purple-to-pink colour variation, the CIELAB a* and b* coordinates were compared with the overall band-position parameter Σ*λ* (Fig. 10(d)). Here, Σ*λ* is defined as the sum of the wavelengths corresponding to the starting point, absorption maximum, and ending point of the approximately 560 nm absorption band; a lower Σ*λ* therefore represents an overall shift of the absorption feature toward shorter wavelengths. The distributions of a* and b* indicate that the two chromatic coordinates vary in a coupled manner with the spectral position of the absorption band rather than following independent monotonic trends. In particular, intervals showing greater variation in a* also exhibit greater variation in b*, indicating coordinated changes in the red–green and blue–yellow components. Because the perceived hue of these samples is determined by the relative balance between positive a* and negative b*, the effect of the spectral shift is more directly expressed by the hue angle *h*° than by either coordinate alone.

Consistent with this interpretation, Fig. 10(e) reveals a significant negative correlation between Σ*λ* and hue angle *h*° (*R*^2^ = 0.871), where Σ*λ* is defined as the sum of the starting point, peak maximum, and ending point wavelengths of the 560 nm absorption band to describe its overall spectral shift. A lower Σ*λ* therefore represents a coordinated shift of the starting boundary, absorption maximum, and ending boundary toward shorter wavelengths. As Σ*λ* decreases, indicating a shift of the absorption band toward shorter wavelengths, *h*° increases and the colour shifts toward pink-purple; conversely, a shift toward longer wavelengths lowers *h*° and produces a more distinctly purple appearance. This spectral displacement modifies the relative balance of the red (+*a*) and blue (−*b*) components, rather than affecting b* independently, thereby driving the systematic hue variation. Furthermore, a similar negative correlation is observed between Σ*λ* and the Al_2_O_3_/(Fe_2_O_3_ + Mn_2_O_3_) ratio (*R*^2^ = 0.845), suggesting that the development of pink hue in sugilite aggregates is closely linked to Al enrichment.

**Fig. 11:**
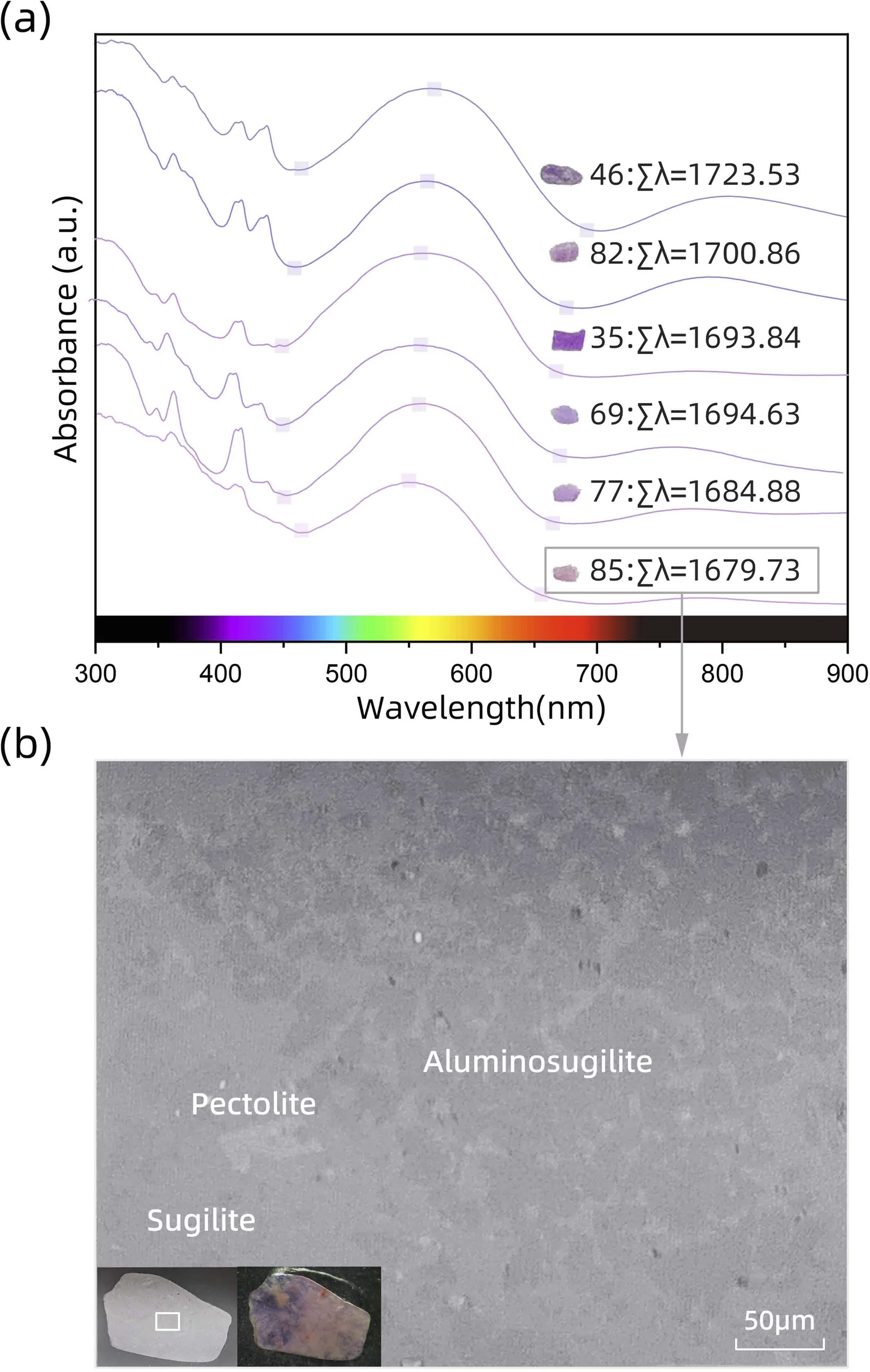
(a) UV-vis spectra of EPMA-analyzed samples arranged in order of decreasing Al_2_O_3_/(Fe_2_O_3_ + Mn_2_O_3_) ratio (from top to bottom); (b) SEM image of sample 85 showing clastic-textured aluminosugilite domains with relatively high Al content.

To minimise the influence of associated minerals on compositional interpretation, six representative samples were further analysed by EPMA, with analytical points selected specifically within continuous sugilite-rich domains free of visible inclusions. The resulting compositions therefore provide a more direct measure of the chemistry of the sugilite phase itself. Table 4 shows that the ratio Al_2_O_3_/(Fe_2_O_3_ + Mn_2_O_3_), calculated from EPMA data, is positively correlated with *h*° (*r* = 0.848), indicating that increasing relative Al_2_O_3_ content within the sugilite lattice shifts the colour toward a pinker hue. Fig. 11(a) presents the UV-vis spectra of these EPMA samples arranged in descending order of Al_2_O_3_/(Fe_2_O_3_ + Mn_2_O_3_). A gradual increase in Σ*λ* can be observed with decreasing Al_2_O_3_ content, indicating an overall red shift of the absorption band.

**Fig. 12:**
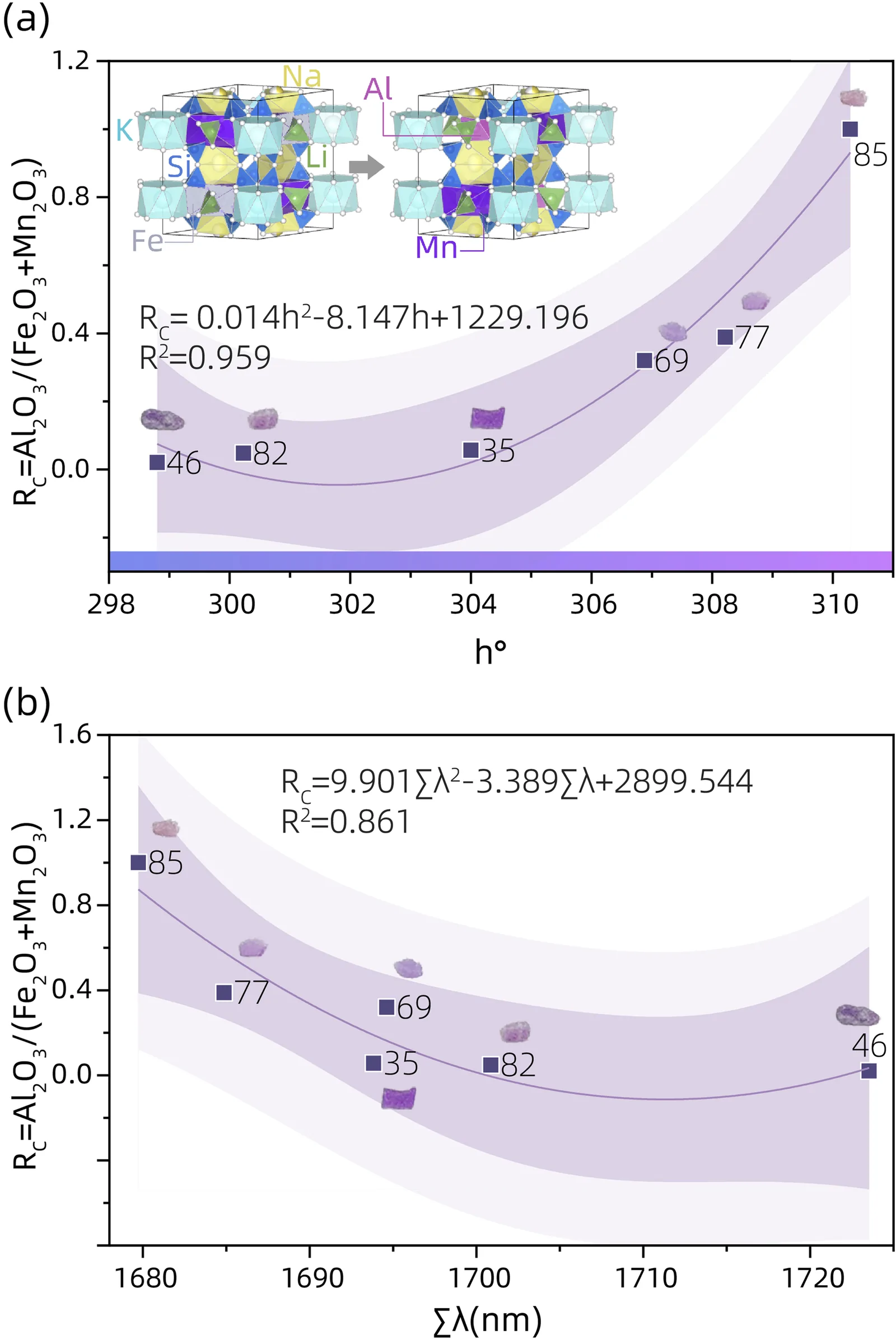
(a) Relationship between hue angle (*h*°) and *R*_C_ = Al_2_O_3_/(Fe_2_O_3_ + Mn_2_O_3_) calculated from EPMA data. The crystal–structure diagram illustrates the substitution of larger Fe^3+^ and Mn^3+^ ions by smaller Al^3+^ ions at the octahedral A site, resulting in lattice compression and modification of the Mn^3+^ crystal-field environment. (b) Relationship between the overall spectral position parameter of the main absorption band (Σ*λ*) and *R*_C_ = Al_2_O_3_/(Fe_2_O_3_ + Mn_2_O_3_), showing the influence of Al substitution on the spectral blue shift of Mn^3+^ absorption.

Fig. 12(a) further demonstrates a highly significant positive correlation between hue angle *h*° and *R*_C_ = Al_2_O_3_/(Fe_2_O_3_ + Mn_2_O_3_) (*R*^2^ = 0.959). Combined with the infrared spectral results shown in Fig. 9(c), increasing substitution of Fe^3+^ and Mn^3+^ by the smaller Al^3+^ ion at the octahedral A site is expected to promote local structural contraction around this site. Because this is an essentially isovalent substitution (Al^3+^ ↔ Fe^3+^/Mn^3+^), the contraction is interpreted primarily as a consequence of the smaller ionic radius of six-coordinated Al^3+^ (0.535 Å) relative to high-spin Fe^3+^ and Mn^3+^ (both approximately 0.645 Å), rather than as a direct effect of charge-compensation processes. The resulting local shortening and strengthening of metal–oxygen bonds may modify the crystal-field environment of neighbouring Mn^3+^. Such local structural contraction may strengthen the crystal field experienced by Mn^3+^, increasing the crystal-field splitting energy (*Δ*_0_). Consequently, higher-energy electronic transitions are favoured, consistent with the observed shift of the principal Mn^3+^ absorption band from approximately 560 nm toward shorter wavelengths. This blue shift results in a gradual colour transition from purple to pink.

Fig. 12(b) shows a negative correlation between Σ*λ* and *R*_C_ = Al_2_O_3_/(Fe_2_O_3_ + Mn_2_O_3_), consistent with the trends obtained from the XRF dataset. Meanwhile, in Fig. 9(c), the integrated infrared characteristic trough parameter Σ*ῡ*(*a* + *b*) increases with increasing relative Al_2_O_3_ content, and the sample sequence is consistent with that observed in Fig. 12(a) and (b). These results further support the mechanism in which Al substitution induces lattice compression and causes a blue shift in the UV-vis absorption band.

Therefore, the pink colour genesis of sugilite aggregates can be explained by two principal mechanisms. First, the concentration of Mn^3+^ controls colour depth and chroma, serving as the primary chromophore. Second, substitution of Fe and Mn by Al modifies the crystal structure and crystal-field environment of Mn^3+^, resulting in a blue shift of the main absorption band and driving the colour transition from purple toward pink.

In addition, SEM observations of sample 85, which possesses the highest relative Al_2_O_3_ content measured by EPMA, reveal that the aluminosugilite domains are darker in colour and display a clastic texture (Fig. 11(b)). The presence of extensive high-Al aluminosugilite regions causes sample 85 to exhibit a lighter and pinker overall appearance compared with ordinary sugilite. This provides further petrographic-scale evidence that high-Al sugilite significantly influences the development of pink colouration.

## 5 Conclusions

This study systematically investigated the colour characteristics, spectroscopic features, and colouration mechanisms of sugilite aggregates using non-destructive analytical methods, including colourimetry, FTIR, Raman, UV-vis spectroscopy, XRF, and EPMA.

Colour clustering analysis demonstrated that Affinity Propagation (AP) clustering outperformed the Gaussian Mixture Model (GMM) in colour classification, providing higher colour homogeneity and lower within-group colour differences. Based on the AP results, four colour grades of sugilite aggregates were established: deep purple, vivid purple, purple, and pastel purple.

FTIR and Raman spectroscopy revealed systematic blue shifts of characteristic vibrational peaks with increasing Al content. Strong correlations between infrared spectral parameters and Al_2_O_3_/(Fe_2_O_3_ + Mn_2_O_3_) ratios indicate that these non-destructive spectroscopic features can be used to evaluate relative Al enrichment in sugilite aggregates. Combined with previous crystallographic constraints and the compositional relationships observed in this study, the investigated Al variation is interpreted as predominantly reflecting the substitution of Al^3+^ for Fe^3+^ and Mn^3+^ at the octahedral A site. Owing primarily to the smaller ionic radius of six-coordinated Al^3+^ relative to Fe^3+^ and Mn^3+^, this essentially isovalent substitution is associated with local structural contraction.

UV-vis spectroscopy confirmed that Mn^3+^ is the principal chromophore responsible for the purple colouration of sugilite aggregates. Increasing Mn concentration enhances both colour depth and chroma through stronger absorption near 560 nm. In contrast, increasing Al substitution is associated with modification of the local crystal-field environment of Mn^3+^ and a blue shift of the principal Mn^3+^ absorption band, thereby shifting the colour from purple toward pink.

These results demonstrate that the colour of sugilite aggregates is jointly controlled by Mn^3+^ concentration and Al-related crystal-chemical modifications. The combined spectroscopic parameters proposed in this study provide a promising non-destructive approach for evaluating relative Al enrichment and colour-related compositional variations in sugilite aggregates. Further calibration using a larger set of compositionally homogeneous reference samples will be required to establish an absolute Al-content detection threshold and develop a fully quantitative gemmological testing framework.

## Author contributions

Pengyu Li: conceptualization, methodology, investigation, data curation, formal analysis, visualization, writing – original draft. Minghong Shi: software, validation, resources. Ye Yuan: software, project administration. Mudan Xu: software, validation. Jiayang Han: data curation, formal analysis. Meilin Zhu: investigation, resources. Jie Hu: data curation, visualization. Ying Guo: supervision, resources, funding acquisition, project administration, writing – review and editing.

## Conflicts of interest

The authors declare that they have no known competing financial interests or personal relationships that could have appeared to influence the work reported in this paper.

## Supplementary Material

RA-016-D6RA04575H-s001

## Data Availability

The data supporting this article have been included as part of the supplementary information (SI). Supplementary information is available. See DOI: https://doi.org/10.1039/d6ra04575h.

## References

[cit1] Armbruster T., Oberhansli R. (1988). Crystal chemistry of double-ring silicates: Structures of sugilite and brannockite. Am. Mineral..

[cit2] Murakami N., Kato T., Miúra Y., Hirowatari F. (1976). Sugilite, a new silicate mineral from Iwagi Islet, Southwest Japan. Mineral. J..

[cit3] Olivier C., Gihwala D., Peisach M., Pineda C. A., Pienaar H. S. (1983). Determination of Lithium in the Gem Mineral Sugilite. J. Radioanal. Nucl. Chem..

[cit4] Dunn P. J., Brummer J. J., Belsky H. (1980). Sugilite, A Second Occurrence: Wessels Mine, Kalahari Manganese Field. Can. Mineral..

[cit5] Roger D. D. , Sugilite and associated metamorphic silicate minerals from Wessels mine, Kalahari manganese field, Master thesis, Universy of Cape Town, Cape Town, South Africa, 1988

[cit6] Dai H. , The Study on Mineral Composition and Color Causes of Sugilite, Master thesis, China University of Geosciences, 2014

[cit7] Zhao B. , Tu C., and Xing G., Sugilite Rock – Testing and Classification [DZ/T 0412—2022 Industry Standard-DZ Geology and Mineral Industry Standard] [Internet]. No. 31, Xueyuan Road, Haidian District, Geological Publishing House, 2022, Beijing, China, http://www.gph.com.cn

[cit8] Wu S., Zhao B., Lu X., Qian W., Lv X. (2023). Study on identification methods of sugilite rock and sugilite rock imitation. Shanghai Institute of Metrology and Testing Technology.

[cit9] Xie Y. (2009). Gemological and Mineralogical Study of Purple Sugilite from South Africa. J. Shenzhen Polytech..

[cit10] Li P., Guo Y. (2025). The impact of mineral composition and trace metal cations on the body color of South African Sugilite Jade. Sci. Rep..

[cit11] Shigley J. E., Koivula J. I., Fryer C. W. (1987). The Occurrence and Gemological Properties of Wessels Mine Sugilite. Gems Gemol..

[cit12] Nagashima M., Fukuda C., Matsumoto T., Imaoka T., Odicino G., Armellino G. (2020). Aluminosugilite, KNa2Al2Li3Si12O30, an Al analogue of sugilite, from the Cerchiara mine, Liguria, Italy. Eur. J. Mineral..

[cit13] Geiger C. A. (2009). A 57Fe Mossbauer spectroscopic study of sugilite, KNa2(Fe3+,Mn3+,Al)2Li3Si12O30. Can. Mineral..

[cit14] Zhu M., Guo Y. (2025). Combined colorimetry and Spectroscopy: A novel nondestructive method for identifying orange garnet species. Spectrochim. Acta, Part A.

[cit15] Ma X., Huang X., Guo Y., Jia Z., Jia S. (2025). Colorimetry Characteristics and Influencing Factors of Sulfur-Rich Lapis Lazuli. Crystals.

[cit16] Della Ventura G., Vigliaturo R., Gieré R., Pollastri S., Gualtieri A., Iezzi G. (2018). Infra Red Spectroscopy of the Regulated Asbestos Amphiboles. Minerals.

[cit17] Kaneva E., Belozerova O., Radomskaya T., Shendrik R. (2024). Crystal chemistry, Raman and FTIR spectroscopy, optical absorption, and luminescence study of Fe-dominant sogdianite. Z. Kristallogr.–Cryst. Mater..

[cit18] Jin S., Renfro N. D., Palke A. C., Ardon T., Homkrajae A. (2025). Application of UV-Vis-NIR Spectroscopy to Gemology. Gems Gemol..

[cit19] Taran M. N., Tarashchan A. N., Rager H., Schott S., Schürmann K., Iwanuch W. (2003). Optical spectroscopy study of variously colored gem-quality topazes from Ouro Preto, Minas Gerais, Brazil. Phys. Chem. Miner..

[cit20] Patel E., Kushwaha D. S. (2020). Clustering Cloud Workloads: K-Means vs. Gaussian Mixture Model. Procedia Comput. Sci..

[cit21] Liu Z., Guo Y. (2022). The Effect of Munsell Neutral Value Scale on the Color of Yellow Jadeite and Comparison between AP and K-Means Clustering Color Grading Schemes. Crystals.

[cit22] Frey B. J., Dueck D. (2007). Clustering by Passing Messages Between Data Points. Science.

[cit23] Tang J., Guo Y., Zhang J. (2023). Colorimetry characteristics and color contribution of fluorescence in natural Cr-containing spinel. Sci. Rep..

[cit24] Hu J., Li P., Yang Y., Yang L., Wang N., Guo Y. (2026). Chemical Controls on the Green Coloration of the Novel Gem Quartzite “Feizhoucui”: A CIE L*a*b* Colorimetric Study. Crystals.

[cit25] Cui L., Guo Y., Tang J., Yang Y. (2023). Spectroscopy Characteristics and Color-Influencing Factors of Green Iron-Bearing Elbaite. Crystals.

[cit26] Shang Y., Guo Y., Tang J. (2022). Spectroscopy and chromaticity characterization of yellow to light-blue iron-containing beryl. Sci. Rep..

[cit27] Pan X., Guo Y., Liu Z., Zhang Z., Shi Y. (2019). Application of cluster analysis and discriminant analysis in quality grading of jadeite red. J. Phys.:Conf. Ser..

[cit28] Jiang Y., Guo Y., Cheung V., Wang P., Westland S., Konys A. (2026). Chrysoprase color grading with machine learning: A systematic approach. PLoS One.

[cit29] Kass R. E., Raftery A. E. (1995). Bayes Factors. J. Am. Stat. Assoc..

[cit30] Curti M. P. H. , Bellerophon Colored Gemstones Color Reference Charts, Bellerophon Gemlab 16 Place Vendome, Paris 75001: Bellerophon Gemlab, 2024, Available from, http://www.bellerophongemlab.com

[cit31] Burns R. G. , Mineralogical applications of crystal field theory: Cambridge University Press, Cambridge Topics in Mineral Physics and Chemistry, New York, 1993, (5), p. 558

[cit32] Handke M., Jastrzębski W. (2005). Vibrational spectroscopy of the double 4-, 6-membered rings in silicates and siloxanes. J. Mol. Struct..

[cit33] Sidorov T. A. (2007). Raman spectra and molecular structure of silicates. Russ. J. Inorg. Chem..

[cit34] Raschke M. B., Anderson E. J. D., Allaz J., Friis H., Smyth J. R., Tschernich R. (2016). *et al.*, Crystal chemistry of brannockite, KLi3Sn2 Si12O30, from a new occurrence in the Golden Horn Batholith, Washington State, USA. Eur. J. Mineral..

[cit35] Dutta P. K., Shieh D. C., Puri M. (1987). Raman spectroscopic study of the synthesis of zeolite Y. J. Phys. Chem..

[cit36] Ferraris C., Pignatelli I., Cámara F., Parodi G., Pont S., Schreyer M. (2020). *et al.*, Laurentthomasite, Mg2K(Be2Al)Si12O30, a new milarite-group-type member from the Ihorombe region, Fianarantsoa Province, Madagascar. Eur. J. Mineral..

[cit37] Reddy B. J., Frost R. L., Martens W. N., Wain D. L., Kloprogge J. T. (2007). Spectroscopic characterization
of Mn-rich tourmalines. Vib. Spectrosc..

[cit38] Gong N., Wang C., Xu S. (2023). Color Origin of Greyish-Purple Tremolite Jade from Sanchahe in Qinghai Province, NW China. Minerals.

[cit39] Lu R. (2012). Color Origin of Lavender Jadeite: An Alternative Approach. Gems Gemol..

[cit40] Vigier M., Fritsch E. (2020). Pink Axinite from Merelani, Tanzania: Origin of Colour and Luminescence. J. Gemmol..

[cit41] Manning P. G. (1968). Absorption spectra of the manganese-bearing chain silicates pyroxmangite, rhodonite, bustamite and serandite. Can. Mineral..

[cit42] Manning P. G. (1969). An optical absorption study of the origin of colour and pleochroism in pink and brown tourmalines. Can. Mineral..

[cit43] RexV G., ThomasJ A., GeorgeR R. (1974). A Spectrographic Interpretation of the Shock-Produced Color Change in Rhodonite(MnSiO3): The Shock-lnduced Reduction of Mn(lll) to Mn(ll). Am. Mineral..

[cit44] Fritsch E., Shigley J. E. (1994). Causes of the purple and pink colours of manganoan sugilites from the Wessels Mine, South Africa. Mineral. Mag..

[cit45] Giester G., Rieck B. (1996). Wesselsite, SrCu[Si4O10], a further new gillespite-group mineral from the Kalahari Manganese Field, South Africa. Mineral. Mag..

[cit46] Scholtzová E., Smrčok Ľ. (2005). On local structural changes in lizardite-1T: {Si4+/Al3+}, {Si4+/Fe3+}, [Mg2+/Al3+], [Mg2+/Fe3+] substitutions. Phys. Chem. Miner..

[cit47] Armbruster T., Oberhansli R. (1988). Crystal chemistry of double-ring silicates: Structural, chemical, and optical variation in osumilites. Am. Mineral..

